# Identification and Assessment of Secondary Metabolites from Three Fungal Endophytes of *Solanum mauritianum* Against Public Health Pathogens

**DOI:** 10.3390/molecules29204924

**Published:** 2024-10-17

**Authors:** Abraham Goodness Ogofure, Sharon Pauline Pelo, Ezekiel Green

**Affiliations:** Department of Biotechnology and Food-Technology, Faculty of Science, University of Johannesburg, P.O. Box 17011, Doornfontein, Johannesburg 2028, South Africa; spelo@uj.ac.za

**Keywords:** fungal endophytes, *Paracamarosporium leucadendri*, *Penicillium chrysogenum*, *Fusarium* sp., LC-QTOF-MS, crude extracts

## Abstract

Fungal endophytes, symbiotic microorganisms residing within plants, are renowned for producing bioactive secondary metabolites with diverse beneficial properties. We investigated the antimicrobial potential of fungal endophytes isolated from *Solanum mauritianum*, an invasive weed, against clinically significant bacterial pathogens. Selected fungal endophytes (*Penicillium chrysogenum*, *Fusarium* sp., and *Paracamarosporium leucadendri*) were isolated from the plant’s leaves and fruits. Their crude extracts were tested against various referenced strains, such as *Mycobacterium* species (*M. smegmatis* ATCC 607 and *M. bovis* ATCC 27290), *Staphylococcus aureus* ATCC 6571, *Bacillus subtilis* ATCC 11774, *Klebsiella* species (*K. pneumoniae* ATCC 10031 and *K. oxytoca* ATCC 8724), *Escherichia coli* ATCC 10536, and *Pseudomonas aeruginosa* ATCC 10145, using the Kirby-Bauer disk diffusion method. Resazurin Microtiter Assay was used for the determination of the minimum inhibitory concentration. The chemical nature of the secondary metabolites in the crude extracts produced by fungal endophytes was evaluated using high-resolution liquid chromatography–mass spectrometry (LC-MS) using water and acetonitrile gradient. Liquid chromatography quadrupole time-of-flight mass spectrometry (LC-QTOF-MS/MS) was employed for untargeted metabolomics. LC-QTOF-MS/MS identified 63 bioactive compounds across the three endophytes. *P. chrysogenum* had the highest activity against *S. aureus* and *M. smegmatis* (1.15 mg/mL and 0.02 mg/mL, respectively), while *P. leucadendri* demonstrated moderate activity against *M. smegmatis* (2.91 mg/mL) and *E. coli* (1.16 mg/mL). *Fusarium* sp. exhibited the broadest spectrum of antibacterial activity, with MIC values ranging from 0.03 mg/mL (*B. subtilis*) to 10 mg/mL (*M. smegmatis*). *P. leucadendri* produced 29 metabolites, *Fusarium* sp. had 23 identified metabolites, and a total of 11 metabolites were identified from *P. chrysogenum*. The fruits of the plant, accounting for 60%, appeared to be the most abundant in the endophyte diversity when compared to the stems and leaves. This study highlights the potential of fungal endophytes from *S. mauritianum* as a source of novel bioactive compounds, particularly against multidrug-resistant pathogens, contributing to the ongoing efforts to combat antimicrobial resistance.

## 1. Introduction

The escalating global burden of antimicrobial resistance (AMR) underscores the urgent need for novel therapeutic agents. AMR is a complex issue driven by the misuse and overuse of antibiotics in human and animal health, leading to the emergence of drug-resistant pathogens [1,2,3,4]. The consequences of AMR are far-reaching, with the potential to reverse decades of progress in healthcare by increasing the rates of morbidity and death, prolonging stay in hospitals, and escalating the cost of healthcare [5,6,7]. To combat this growing threat, the development of new antimicrobial compounds is imperative, and natural product discovery, particularly from underexplored microbial sources, has emerged as a promising avenue for addressing this crisis.

Microbial endophytes, dwelling asymptomatically inside plant tissues, constitute a rich store of chemically varied secondary metabolites with potential antibacterial capabilities and strong biological activity [8,9,10]. Recent advancements in microbial and plant interaction studies have highlighted fungal endophytes as a significant reservoir of bioactive compounds, making them a focal point in the search for new antimicrobial agents amidst escalating antibiotic resistance [11,12,13]. Endophytes, which form mutualistic associations with their host plants, are known for their extensive production of secondary metabolites that exhibit a wide range of biological activities [14].

The plant *Solanum mauritianum*, often regarded as an invasive species, is an overlooked medicinal plant that harbors a rich diversity of fungal endophytes [10]. This plant, belonging to the *Solanaceae* family, is widely distributed across tropical and subtropical regions, including the biodiverse ecosystem of Johannesburg, South Africa. Despite its invasive status, *S. mauritianum* has garnered interest in ethnopharmacology due to its traditional medicinal uses and potential therapeutic properties [10,15]. However, the endophytic fungal community associated with this plant and the bioactive compounds it produces remain largely unexplored.

This study aims to bridge the knowledge gap by investigating the bioactive components (secondary metabolites) produced by the endophytes selected from different parts of *S. mauritianum* in Johannesburg, South Africa. This research is focused on the detection, isolation, and characterization of novel bioactive compounds from three fungal endophytes with potential applications against public health pathogens. By leveraging advanced analytical techniques and bioassay-guided fractionation, we seek to uncover promising lead compounds that could address the pressing need for new antimicrobial agents. This study further aims to investigate the assortment of secondary metabolites by the selected endophytic fungi associated with *S. mauritianum* in Johannesburg and evaluate their potential as producers of antibacterial compounds. By focusing on this invasive plant species, we seek to uncover previously unexplored microbial resources that could contribute significantly to the development of new antibiotics against public health pathogens, including drug-resistant strains of bacterial species.

## 2. Results and Discussion

### Isolation and Distribution of Fungal Endophytes

The isolation, distribution, and total secondary metabolites of the fungal endophytes (*Penicillium chrysogenum*, *Fusarium* sp., and *Paracamarosporium leucadendri*) in different parts of the *S. mauritianum* plant are shown in Table 1. *Paracamarosporium leucadendri* was exclusively found in the fruits of the plant, while *Fusarium* sp. and *Penicillium chrysogenum* were both found in the leaves and fruits. None of the endophytes were found in the stem of the *S. mauritianum* plant. These results were found to be consistent with the reports of Torta et al. [16] and de Siqueira et al. [17], who opined that endophytic colonization occurs mainly in the roots, leaves, and fruits rather than in other parts of the plant. However, according to de Siqueira et al. [17], there was less endophytic colonization of the stem compared to the leaves and other parts of the plants. The results from this study also indicate a tissue-specific colonization pattern for the identified endophytes where *Paracamarosporium leucadendri* shows some predilection for the fruits (maybe for some specific function in the general plant’s well-being), and both *Fusarium* sp. and *Penicillium chrysogenum* are conspicuously present in the leaves and fruits of the plant. The phenomenon of tissue-specific colonization by endophytes was also similarly reported by Behie et al. [18], who revealed that the endophytic fungus *Metarhizium* spp. showed preferential colonization/localization for certain plant tissues. The tissue-specific colonization of endophytes is a testament that the endophytes may be hyperdiverse because of their host preference and colonization patterns in healthy plants [9,19]. This information is crucial for understanding the tissue-specific colonization of fungal endophytes in *S. mauritianum*, which can be important for studies on plant–endophyte interactions, potential bioactive compound production, and ecological research on fungal distributions in plants. The total secondary metabolites isolated from three different endophytic fungi (*Fusarium* sp., *P. chrysogenum*, and *P. leucadendri*) revealed that *P. leucadendri* produced 29 secondary metabolites, *Fusarium* sp. produced 23 secondary metabolites, and *P. chrysogenum* had the fewest total metabolites at 11.

**Figure 1:**
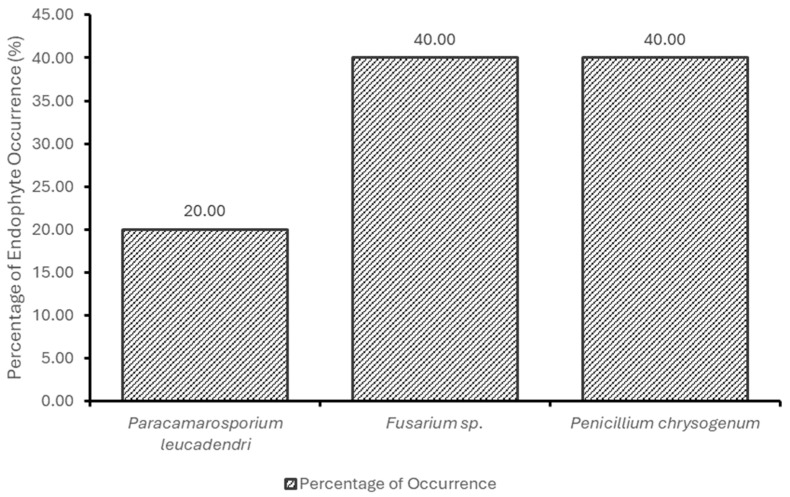
Percentage of occurrence of fungal endophytes in *S. mauritianum*.

**Figure 2:**
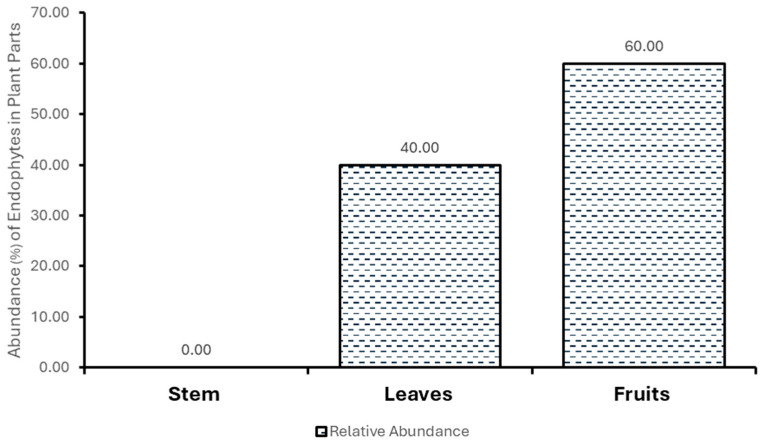
Relative abundance (%) of fungal endophytes in *S. mauritianum* plant parts.

The percentage of occurrence of fungal endophytes in *S. mauritianum* and the relative abundance (%) of fungal endophytes in *S. mauritianum* plant parts are shown in Figure 1 and Figure 2. *Penicillium chrysogenum* and *Fusarium* sp., the most occurring fungal endophytes, are obtained from most plant parts of *S. mauritianum*, while the fruits of the plants appear to be the parts most rich in endophyte diversity (in regard to the stems and leaves). This finding is inconsistent with the findings of Yu et al. [20], who opined that the leaves are the richest with endophytes compared to the fruits of the *Camellia oleifera* plant. This inconsistency could be due to differences in plant species, environmental interference, and methods employed for the isolation of endophytic microorganisms. In maize plants and woody plants, the leaves have been touted as the most endophyte-rich parts of the plants [20,21]. In this study, *P. chrysogenum* and *Fusarium* sp. were the most frequently occurring fungal endophytes from *S. mauritianum*, while the fruits and then the leaves had the highest relative abundance of fungal endophytes in the invasive plant species. Almost all plant species harbor fungal endophytes, which can asymptomatically colonize different parts of the plants, including the roots, stems, leaves, branches, and fruits [22].

**Figure 3:**
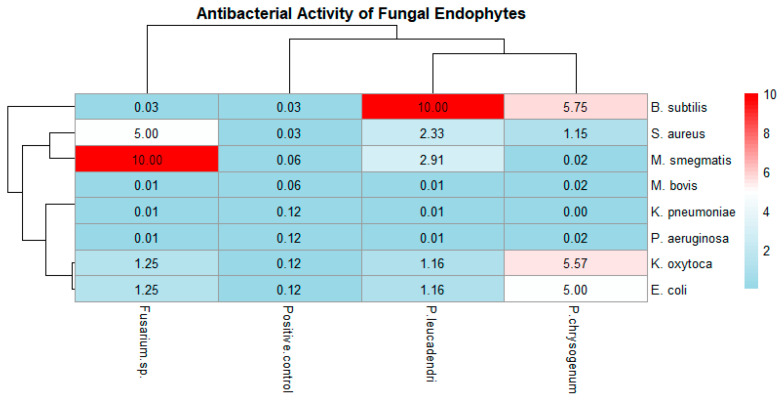
Pheatmap showing antibacterial activity/effectiveness of crude fungal endophytes against pathogens of public health importance. *B. subtilis* (1), *S. aureus* (2), *K. oxytoca* (3), *K. pneumoniae* (4), *E. coli* (5), *P. aeruginosa* (6), *M. bovis* (7), and *M. smegmatis* (8). The colors of each cell indicate the level of antibacterial activity measured by their respective MICs. The positive control was Rifampicin (40 µg/mL), and the crude extracts of fungal endophytes are presented in mg/mL.

The pheatmap (Figure 3) illustrates the antibacterial activity and MIC (mg/mL) of the crude extracts of fungal endophytes against bacterial pathogens of public health importance. “Red” denotes no antibacterial activity, indicating less effective antibacterial activity; “Light blue” has lower MIC values, indicating more effective antibacterial activity; and “white” indicates intermediate MIC values. Furthermore, the heatmap shows the similarities in antibacterial activity patterns between different endophytes, with row clusters revealing that *P. chrysogenum* and *Fusarium* sp. have distinct activity profiles, while *Paracamarosporium leucadendri* clusters closer to *Fusarium* sp. The column cluster suggests that endophytes impact diverse bacterial pathogens in comparable ways. *B. subtilis* and *S. aureus* exhibit unique sensitivity patterns in comparison to other infections. *Penicillium chrysogenum* is the most effective endophyte against *B. subtilis* and *S. aureus*. *Paracamarosporium leucadendri* is somewhat active against *E. coli* and *M. smegmatis*. *Fusarium* sp. had the highest antibacterial efficacy against the investigated pathogens (given its low MIC values).

These findings were found to be consistent with several reports on the antibacterial properties of fungal endophytes against Gram-positive and -negative bacteria alike [3,9,11,23,24]. Secondary metabolites from *Fusarium* sp. in this study, like the other fungal endophytes, were found to have broad-spectrum activity against bacterial pathogens of importance to public health, including *Mycobacterium* sp. This activity may be due to the presence of the antitubercular compound (Tubulysin B) identified in the crude extract of the fungal endophyte. Consistent with the findings mentioned above about *Fusarium* sp., Gordien et al. [25] and Pelo et al. [9] reported that fungal endophytes from Scottish plants and an invasive weed from Johannesburg showed antibacterial activity against different *Mycobacterium* strains with respective MIC values in the ranges of 2–8 mg/mL and 0.03–4 mg/mL, which are comparable to the findings in our study. MIC values for crude extracts less than 0.07 mg/mL are usually considered promising for further investigation following breakpoints for most antibiotics [26]. The literature is replete with information about fungal endophytes against pathogens such as *S. aureus* and *E. coli* and their corresponding antibacterial efficacy [6,13,24]. *P. chrysogenum* was found to have the best activity against *B. subtilis*, as *Fusarium* sp. was found to have little or no antibacterial effect against the bacterial pathogen. This finding was consistent with the report by Kyekyeku et al. [27], who opined that no antibacterial activity was observed for the endophytic fungus *Fusarium solani.* However, considerable activity was found against other bacterial pathogens tested in the study, as was observed in the current study.

The proportion of identified secondary metabolites provides insights into the potential utility of these fungal endophytes in developing pharmacologically active compounds of importance in medicine and human health. Overall, *Fusarium* sp. exhibited the highest number of total secondary metabolites, while *P. leucadendri* and *P. chrysogenum* had fewer total metabolites than *Fusarium* sp. The screening technology employed for the fungal endophytes in this study revealed promising secondary metabolites of medical and agricultural importance [28]. An untargeted approach (LC-QTOF-MS) was used to evaluate the crude extracts of *Fusarium* sp., *P. chrysogenum*, and *P. leucadendri*, and 87 (in total) bioactive compounds were found. This report is the first of its kind to evaluate nontargeted compounds for the three fungal endophytes. Indeed, there needs to be more research on secondary metabolites and their potential pharmacological and health benefits. In the literature, there are no current reports about the bioactive compounds of *P. leucandendri*, except for the report by Pelo et al. [9]. Some of the bioactive compounds identified in this study for fungal endophytes have been revealed to have ecological, agricultural, pharmacological, and medicinal importance.

The compounds 3-allyl-thiophene, 4-vinyl isoquinoline, 2-nitroimidazo[2,1-b][1,3]oxazole, and Withasomnine from *Fusarium* sp. have been reported to have antibacterial activity against different pathogens of public health significance [29,30,31,32]. Crude extracts from *Fusarium* sp. have been reported to have antibacterial activities against pathogens of both plants and animals, with the secondary metabolite aurofusarin (a homodimeric naphthoquinone) being a prominent compound [33]. Interestingly, the secondary metabolite aurofusarin was not isolated in this study as a testament to the fact that the production of secondary metabolites by fungal endophytes is a function of several factors, which include the methods and solvents used for extraction, growth media, and other optimization parameters. To further buttress this point, Lopes et al. [34] reported different antimicrobial efficacy for the same fungus grown or fermented using different substrates, with greater antimicrobial activity observed for cheese whey cultures compared to agro-waste substrates.

Similarly, fusarithioamide (an antimicrobial compound) identified from *Fusarium* sp. was reported by Ibrahim et al. [35] to have both antibacterial and antifungal properties. Since the discovery of penicillin, *P. chrysogenum* has been in the limelight, and there have been non-stop revelations about secondary metabolites of importance to plants and humans in that regard. Several studies have shown that secondary metabolites from *P. chrysogenum* contribute immensely to anti-cancer and antimicrobial activities [36,37]. Similar to the results obtained in this study for secondary metabolites of *P. chrysogenum*, El-Sayed et al. [38] and Gao et al. [39] opined that not only do the metabolites have pharmacological activities, but they are also derivatives of polyketide, glycerol, and monoterpene compounds. Some of the secondary metabolites identified in this study appear to be novel in the context of being reported for the first time as being produced by the fungal endophytes (*P. leucadendri*, *Fusarium* sp., and *Penicillium chrysogenum*) examined in this study. While a few of these compounds have been previously reported in the literature, they were associated with other microorganisms, such as bacteria or fungi, and not with the specific fungal endophytes studied in this work.

The novel secondary metabolites from *P. leucadendri*, *Fusarium* sp., and *Penicillium chrysogenum*, which include L-Cladinose, albaflavenone, tridemorph, carvacrol, 6-*cis*-docosenamidse, seiricardine A, and rishitin, amongst others, have been individually reported in other studies to be produced from diverse microbial sources, and these compounds were reported to have antimicrobial properties [40,41,42,43,44,45,46,47,48,49,50,51]. Tridemorph is a morpholine-based compound which has demonstrated potent antifungal activity. The MS/MS analysis showed a precursor ion at *m*/*z* 298 (Supplementary Material S/N 17b). One prominent fragmentation event was the loss of water (H_2_O), resulting in a fragment ion at *m*/*z* 280. This water loss is typical for morpholine derivatives, and the further cleavage of the morpholine ring produced a fragment at *m*/*z* 166, which is characteristic of the breakdown of the morpholine structure [52,53]. Albaflavenone, a sesquiterpene, was also detected and identified from the precursor ion in the MS/MS spectrum, where it appeared at *m*/*z* 219. The fragmentation analysis revealed a key loss of water (H_2_O), leading to a fragment at *m*/*z* 201, which is a common fragmentation pattern for sesquiterpenes [54]. One of the sesquiterpene rings was further cleaved, resulting in a fragment ion at *m*/*z* 177, thereby confirming the presence of the tricyclic structure and supporting its antimicrobial potential [54] (Supplementary Material S/N 25). The activity of albaflavenone has been reported by Lin and Cane [46] and Huang et al. [47] to be effective against a plethora of microbial species, but the metabolite was a product of *Streptomyces coelicolor* and *Dictyophora indusiata*, respectively. There are several reports of the antibacterial substance albaflavenone in the literature [46,47,48,49], and this is one of the foremost studies which revealed that the compound can be obtained from *P. leucandendri.* L-Cladinose was also another secondary metabolite produced by the fungal endophyte *P. leucandendri* in this study. Reports revealed that this compound has apotent antibacterial activity similar to macrolide antibiotics [40,41]. There have also been reports of antimicrobial activity in 6-*cis*-docosenamidse, seiricardine A, and rishitin against microbial pathogens of public health importance. However, none of the reports have shown any of these metabolites to be produced by *P. leucandendri*, save the aforementioned report by Pelo et al. [9]. Aside from the antibacterial activity of these secondary metabolites, Sobrinho et al. [51] also revealed that some of these metabolites have antifungal activity against dermatophytes (*Trichophyton* and *Microsporum*). Interestingly, aside from the fact that these metabolites have antibacterial activity, as reported in this study, Yoshioka et al. [50] opined that rishitin could be employed for the control of plant pathogens. Furthermore, other studies showed that the secondary metabolites produced by these fungal endophytes are sometimes produced in response to the presence of phytopathogens to help the plant withstand the threats and subsequent colonization by the phytopathogens [42,43,44]. Fecosterol was also identified in *P. leucandendri.* For this compound, an MS/MS analysis revealed a precursor ion at *m*/*z* 399.635, and one of the prominent fragmentation events was the loss of a water molecule (H_2_O, mass = 18), resulting in a fragment ion at *m*/*z* 381. This loss of water is a common characteristic of sterols and has been reported in previous studies [55,56]. The subsequent cleavage of the steroid ring produced a fragment ion at *m*/*z* 149, consistent with known patterns of sterol degradation in mass spectrometry [56]. These fragmentation patterns support the structural identification of Fecosterol and its potential role in antimicrobial activity (Supplementary Material S/N 3).

The secondary metabolites of *P. leucadendri*, *Fusarium* sp., and *P. chrysogenum* from *S. mauritianum* shown above in Table 2, Table 3 and Table 4 revealed a plethora of metabolites showing their retention times, *m*/*z* values, and chemical formulas. A comprehensive list of the 63 untargeted compounds from these endophytes is shown in the tables above. The MS data for the identification of the compounds are presented in the Supplementary Material, with the retention time, mass-to-charge ratio, and chemical formula for all compounds presented in Table 2, Table 3 and Table 4.

Rishitin, a sesquiterpenoid phytoalexin, was also detected and identified in fungal endophyte. The MS/MS analysis identified a precursor ion at *m*/*z* 223, and a key fragmentation event was the loss of a water molecule, leading to the formation of a fragment at *m*/*z* 205. This is consistent with the loss of hydroxyl groups commonly observed in sesquiterpenoids [57]. Further fragmentation resulted in the cleavage of the hydrocarbon chain, producing a fragment ion at *m*/*z* 149, confirming the breakdown of the terpenoid structure [57,58]. These findings correlate with rishitin’s known antimicrobial action, likely due to its ability to disrupt microbial cell membranes [59] (Supplementary Material S/N 29).

Several of the novel antimicrobial compounds (secondary metabolites) identified from the endophytes in this study, such as rishitin, carvacrol, 6-*cis*-docosenamide, and seiricardine A from *P. leucandendri*, have previously been reported to have potent antibacterial activity against a wide range of bacterial pathogens of significant public health concern. These specialized metabolites are known to exhibit broad-spectrum antimicrobial properties, contributing to their potential as promising candidates for addressing antibiotic resistance and infectious diseases [60,61,62]. While it has been theorized that plant endophytes are repositories of bioactive specialized or secondary metabolites with structural and chemical diversity [60], there is a shred of evidence that rishitin has been used in the past against plant diseases such as the late blight and early blight of potato caused by *Phytophthora infestans* and *Alternaria solani*, respectively [61]. Rishitin, a phytoalexin, was reported to be synthesized after 14 h of post-inoculation of tomato (*Solanum lycopersicum*) root by the fungus *Pythium oligandrum* [62], and Mili [60] opined that the compound (rishitin) is a plant-derived compound of *Solanum* species reported to be produced by endophytic fungi. There are pieces of evidence showing that the spectrum of activity of the compound rishitin could be broad in that it was able to inhibit the growth of bacteria and fungi, as reported in the aforementioned studies. Carvacrol, a monoterpenoid phenol, is one of the specialized metabolites identified from *P. leucandendri* in this study. The MS/MS fragmentation of carvacrol begins with the precursor ion at *m*/*z* 151; the loss of a methyl group (*m*/*z* 135) and further cleavage of the isopropyl chain (*m*/*z* 107) help to confirm its identity and structural stability (Supplementary Material S/N 26). Carvacrol has also been reported in fungal endophytes isolated from the leaves and stems of Thymus species. This compound is well known for its antimicrobial properties, further emphasizing its potential significance in the arsenal of bioactive metabolites with promising therapeutic applications [63,64]. Carvacrol has demonstrated broad-spectrum antimicrobial activity against a variety of bacterial and fungal isolates of public health and food security importance [65,66,67]. Sayed et al. [64] suggested that the antimicrobial potency of crude extracts from *Thymus vulgaris* and its associated endophytes may be attributed to the presence of carvacrol, further supporting its role as a key bioactive compound in these extracts. Unlike rishitin, carvacrol has been isolated from a variety of plants beyond Solanum species.

In contrast, the secondary metabolite 6-*cis*-docosenamide has limited presence in the literature, with only a few mentions, such as in the report by Elkhateeb et al. [68]. This compound was among the many secondary metabolites isolated from *Ganoderma applanatum* that have been shown to induce apoptosis through various pathways. However, a variant or isomer of this compound, 13-docosenamide, has been reported by Dos-Reis et al. [69] to have broad-spectrum antibacterial properties, further expanding the chemical diversity and potential biological activity within this class of molecules. The compound seiricardine A has been reported to be a novel compound produced by endophytic fungi, and this compound has been reported to have broad-spectrum antimicrobial activity against bacteria and fungi [70,71,72]. The secondary metabolites were reported to be produced by different fungal endophytes, which include *Cytopsora* sp. (from Chinese mangrove plant) [70], *Aspergillus* and *Trichoderma* species [72], and *Pestalotia* sp. isolated from the leaves of *Heritiera fomes* [71].

The novel secondary metabolites identified from *Fusarium* sp., which include 3-allyl-thiophene, 2-nitroimidazo[2,1-b][1,3]oxazole, Withasomnine, 1-(4-methyl-1-piperidinyl)-2-tetra decanol, and 4-dodecyl morpholine, to mention a few, have been scarcely reported in the literature, especially the former. While there are no reports of the exact compound (3-allyl-thiophene), there are reports of its derivatives and parent compound thiophenes. Recent studies have shown the compound to be produced by two endophytic *Penicillium* species (*P. lanosum* and *P. radiatolobatum*) [73]. Ibrahim et al. [74] opined that thiophenes are naturally occurring specialized metabolites in plants; however, the presence of crude extracts in *Fusarium* sp. in this study can be attributed to biosynthetic function. Other specialized metabolites from *Fusarium* sp., which are 2-nitroimidazo[2,1-b][1,3]oxazole, Withasomnine, 1-(4-methyl-1-piperidinyl)-2-tetra decanol, and 4-dodecyl morpholine, have also scarcely been reported in recent studies. However, while the exact compounds identified in this study (except for withasomine) have not been reported, their derivatives or parent compounds have been shown to be produced by fungal endophytes and even chemically synthesized in some instances. Withasomine, as reported by Gowtham et al. [32] and George et al. [75], is a unique alkaloid with a broad range of antimicrobial activity naturally produced by the Indian medicinal herb *Withania somnifera*. Zhuang et al. [76] reported that nitroimidazole-oxazolidinone, a compound from which 2-nitroimidazo[2,1-b][1,3]oxazole can be derived, shows some synergistic activity against anaerobic pathogens, and thus, it is a vital tool in the fight against antimicrobial resistance. Variants or isomers of the novel secondary metabolite (1-(4-methyl-1-piperidinyl)-2-tetra decanol) have been identified and reported by Vinaya et al. [77] (as piperidinyl tetrahydrothieno[2,3-c]isoquinolines) and Zaki et al. [78] (as 1-benzhydryl-sulfonyl-4-(3-(piperidin-4-yl) propyl)piperidine) to have a broad spectrum of activity against a plethora of microbial isolates. Similarly, variants of the compound 4-dodecyl morpholine have been reported to have antibacterial activity against bacterial pathogens [79,80]; however, none of the reports have shown that fungal endophytes produced the compound.

Some of the novel antibacterial compounds of endophytic *Penicillium chrysogenum* from *S. mauritianum* in this study are 1,8-Diazacyclotetradecane-2,9-dione, L-Ascorbic acid, 6-octadecanoate, Tubulysin B, and Lunacrine. There is limited information or studies about 1,8-Diazacyclotetradecane-2,9-dione as a secondary metabolite with antibacterial activity. Pelo et al. [9] identified the compound from crude extracts of *S. mauritianum*, while Zhu et al. [81] identified the metabolites from the interaction between *Phytophthora* sp. and *Solanum tubersosum*. A derivative of the compound L-Ascorbic acid, 6-octadecanoate, has been reported to be produced by some bacterial endophytes of *Ocimum sanctum* and *Artemisia nilagirica* with activity against the plant pathogenic fungus *Fusarium oxysporum* [82] and bacterial pathogens like *S. aureus* [83]. Gram-negative bacterial isolates have been reported to produce Tubulysins A and B with antibacterial activity against multidrug-resistant pathogens [84].

Furthermore, the secondary metabolite, Tubulysin, was reported to have broad microbial activity even against viruses [85]. Lunacrine has also been reported to have antibacterial and antifungal properties, but the metabolite was isolated from the bark of the Lunas-bargon plant. More recently, the compound was also identified to have both antimicrobial and cytotoxic properties [86].

## 3. Materials and Methods

### 3.1. Study Sites and Sample Collection

This study was conducted in Johannesburg, where *Solanum mauritianum* plants were collected from various points in the city. Healthy leaves, fruits, and stems of *S. mauritianum* were carefully excised and placed in sterile plastic bags [45]. A botanist from the University of Johannesburg identified the samples, and a voucher specimen was deposited in the university’s herbarium. Transportation of the samples to the laboratory was carried out on ice, and processing of the samples was carried out within 24 h. of collection to ensure the viability of the endophytes.

### 3.2. Isolation of Fungal Endophytes

The plant samples, which were obtained in five independent replicates, underwent a rigorous surface sterilization protocol for the elimination of epiphytes. Samples were sequentially immersed in ethanol (70%) for 60 s, 2% solution of sodium hypochlorite (bleach) for 5 min, and then sterile distilled water (for proper rinsing to remove disinfectants) [87]. Following sterilization and drying, small sections (5 mm pieces) were aseptically excised and inoculated onto chloramphenicol-amended SDA (Sabouraud Dextrose Agar), PDA (Potato Dextrose Agar), and NA (Nutrient Agar) for fungal isolation. Plates were incubated at 25 °C for 7–14 days, and emerging fungal colonies were subsequently purified through subculturing on fresh PDA and SDA plates.

### 3.3. Identification of Fungal Endophytes

The selected endophytic fungi were identified through a combination of morphological and molecular techniques. Colony morphology, spore structure, attachments, and pigmentations were examined microscopically in addition to macroscopic features like the mycelium color on the plate, reverse plate color characteristics, and growth pattern [88]. Three of the pure fungal colonies of interest were selected for molecular identification. Molecular identification was achieved by successful fungal DNA extraction, amplification, and sequencing of the ITS region of the fungal rDNA [89,90,91], followed by BLAST comparison against the NCBI GenBank database [89]. Briefly, the fungal mycelium from a 3-day-old pure culture grown in PDA broth was aseptically pipetted into 2 mL Eppendorf tubes after harvest from the PDA plates using an inoculation loop. The mycelial mat was pelleted by centrifugation at 13,000× *g* for 5 min and washed twice with Tris-EDTA (TE) buffer. After decanting the TE buffer following the second spin, 300 µL of extraction buffer was added to the tubes. The pellets were then flash-frozen and ground into a fine powder. This material was lysed using CTAB extraction buffer and β-mercaptoethanol, followed by incubation at 65 °C. Phase separation was achieved using chloroform/isoamyl alcohol, and the aqueous phase containing DNA was isolated. DNA was precipitated using cold isopropanol, pelleted by centrifugation, and washed with 70% ethanol. After air-drying, the DNA pellet was rehydrated in TE buffer.

The extracted DNA quantity and quality were evaluated using agarose gel electrophoresis and spectrophotometry. Following DNA extraction, two primer sets (ITS4 (5′ to 3′) (TCCTCCGCTTATTGATATGC) and ITS1 (5′ to 3′) (TCCGTAGGTGAACCTGCGG)) were used to amplify the ITS target region accordingly. Extraction of the PCR products from the gel was carried out using a Gel DNA Recovery Kit (Zymo Research Zymoclean™, Irvine, CA, USA). Lastly, the purified PCR products were sequenced on a ABI PRISM™ 3500xl Genetic Analyzer (ThermoScientific, Waltham, MA, USA) in both forward and reverse directions [89,90,91]. Representative sequences for each endophytic fungal isolate were deposited in GenBank. Reference taxa species were identified as those with 99% ITS sequence similarities, and some species were identified at the generic level.

### 3.4. Extraction of Secondary Metabolites from Fungal Endophytes

The extraction of secondary metabolites from fungal endophytes was carried out following a comprehensive dual-solvent process. The endophytes were initially cultivated on potato dextrose agar, from whence 10 mm plugs were excised and transferred to 200 mL bottles containing 100 mL of PDB (potato dextrose broth). The fungal cultures were then incubated statically for 14 days at 25 °C ± 2 °C to ferment according to the protocol described by Marcellano et al. [92]. After fermentation, two distinct phases were employed in the extraction procedure. In the first phase, the dry mycelial mat formed on the surface was carefully macerated using a pestle and mortar before filtration through a filter paper (Whatman No. 1). Shortly after that, ethyl acetate (100 mL) was added to the filtrate and allowed to stand in a separation flask for 24 h at 25 ± 2 °C (room temperature). The resulting organic phase was separated and concentrated at 40 °C under a vacuum utilizing a Labtech EV311H rotary evaporator. This process was repeated three times to ensure the thorough extraction of secondary metabolites. The concentrated extract was then transferred to a McCartney bottle and dried under a laminar flow hood. In the second phase, the dried mycelia and spores underwent further extraction using methanol (100 mL) and were left for 24 h at room temperature. After filtration, this methanol extract was evaporated with agitation at 200 rpm at 40 °C. This dual-solvent extraction method ensures that both the polar and non-polar secondary metabolites from the fungal endophytes were comprehensively extracted to yield a robust extract suitable for antibacterial activity testing.

### 3.5. Inoculum Preparation

The bacterial inocula which were used in this study were prepared using a standard protocol delineated by Marcellano et al. [92], where cultures of *S. aureus* ATCC 6571, *B. subtilis* ATCC 11774, *K. pneumoniae* ATCC 10031, *K. oxytoca* ATCC 8724, *E. coli* ATCC 10536, and *P. aeruginosa* ATCC 10145 were sub-cultured on MHB (Mueller–Hinton broth) and incubated at 37 °C for 18–24 h. The broth cultures were adjusted to correspond to 1.5 × 10^8^ cells/mL (0.5 McFarland Standard) using a UV spectrophotometer. At the same time, a unique method was employed for inocula preparation of the *Mycobacterium* species (*M. smegmatis* ATCC 607 and *M. bovis* ATCC 27290). The bacteria were inoculated into a separate 10 mL broth (Middlebrook 7H9 broth supplemented with Middlebrook OADC (Oliec albumin Dextrose Catalase) growth supplement (Sigma-Aldrich, St. Louis, MO, USA)), which was freshly prepared. The broths were incubated at 37 °C for 7 days (for *M. bovis*) and at 37 °C for 24 h (for *M. smegmatis*), respectively.

### 3.6. Screening of Crude Extracts from Endophytes for Antibacterial Activity

The dried crude extracts were dissolved in 10% DMSO (dimethyl sulfoxide) at a concentration of 10 mg/mL and inoculated onto 6 mm sterile disks, which were allowed to dry for 60 s under aseptic conditions. All tests were conducted in three independent replicates, and the negative control was the 10% DMSO, while the positive control employed was 40 µg/mL of rifampicin antibiotics. The pathogenic bacteria (reference strains) used as the test organisms were cultured on MHA (Mueller–Hinton agar) plates. Incubation of the plates was carried out for 24 h at 37 °C, except for *M. bovis*, which was incubated for seven days following the standard culture protocol described above. The antibacterial activity was assessed by measuring the diameters of the clear zones of inhibition around the disks.

### 3.7. Minimum Inhibitory Concentration (MIC) of Crude Extracts from Endophytes

Resazurin Microtiter Assay (REMA) was employed for the determination of the MIC using the technique explained by Teh et al. [93]. Fresh inocula were prepared from MHA plates into MHB and standardized to a 0.5 McFarland standard. A 1:10 dilution of the inoculum was achieved, and 100 µL of the broth was dispensed into each well of a 96-well flat-bottom microtiter plate (Becton Dickinson, Franklin Lakes, NJ, USA). Serial dilutions of the obtained crude extracts of the endophytic fungi were directly placed into the wells with varying concentrations ranging from 0.0039 mg/mL to 5 mg/mL. Each well then received 100 µL of the bacterial test organism suspension. Both negative and positive controls were included for each isolate, with positive controls ranging from 0.019 µg/mL to 40 µg/mL. Evaporation of the extract during incubation was minimized by the addition of sterile water to the perimeter wells. The plates were sealed in plastic bags, and incubation was carried out for 18–24 h at 37 °C. At the end of the incubation period, resazurin solution (30 µL) was added to each well to indicate bacterial growth. Additional incubation time for up to seven days was required for *M. bovis* before the addition of resazurin. A change in color after the addition of resazurin solution from blue to pink is indicative of bacterial growth. The minimum inhibitory concentration was therefore taken as the least concentration where no color change occurred, or at least the change in color was prevented.

### 3.8. GC-HRTOF-MS (Gas Chromatography High-Resolution Time-of-Flight Mass Spectrometry) Analysis

Into 1 mL of analytical-grade methanol, a weighed portion (I mg) of the crude secondary metabolite was dissolved and vortexed thoroughly before filtration using a 0.2 µm syringe filter. Autosampler vials were immediately used to receive the filtered samples for analysis. GC-HRTOF-MS (LECO Corporation, St. Joseph, MI, USA) was employed for the laboratory analysis, with calibration performed prior to use. The subsequent analyses were conducted using a Pegasus GC-HRTOF-MS instrument (same model) coupled with an Agilent 7890A gas chromatograph (Agilent Technologies, Wilmington, NC, USA). The system was equipped with a multipurpose autosampler (Gerstel MPS from Gerstel Inc., Mülheim an der Ruhr, Germany) and operated in high resolution. The column utilized was a 30 m × 0.25 mm ID × 0.25 µm Rxi^®^-5 ms column (Restek, Bellefonte, PA, USA). The carrier gas used in the analysis was helium, and the rate of flow was 1 mL/min. An injection volume of 1 µL per sample was used in the analysis, and the sample injection was in splitless mode. The transfer line and inlet temperatures were maintained at 225 °C and 250 °C, respectively. The oven temperature program began at 70 °C and held for 0.5 min, and then increased to 150 °C at a rate of 10 °C per minute and held for 2 min, and finally raised to 330 °C at a rate of 10 °C per minute and maintained for 3 min. Electron ionization was performed at 70 eV, and the MS data acquisition rate was set to 13 spectra per second with an *m*/*z* range of 30–700. LECO Chroma TOF-HRT software (version 4.30) was used to process the data obtained from the GC-HRTOF-MS analysis. Notable metabolomic libraries, such as Feihn Maillib and NIST, were used to match the peaks and mass spectra produced. Metabolites were identified and assigned names when the SV (similarity value) exceeded 70% [10].

### 3.9. LC-QTOF-MS Settings

The QToF Series 1.9 (LC-QTOF-MS Compass) Bruker Instrument system (Impact II, Bruker Daltonics GmbH, Baden-Württemberg, Germany) was used to identify the crude extracts from the fungal endophytes. A RESTEC model reverse-phase C18 column (50 mm by 2.1 mm and 1.7 µm particle size) was used to conduct chromatographic separation with 0.1% formic acid in acetonitrile and water constituting the mobile phase. The mobile phase consisted of 0.1% formic acid in water and acetonitrile. Mass spectrometry data were obtained using electron spray ionization (ESI), scanning between 50 and 1600 *m*/*z* at a nebulizer pressure of 1.8 bar, and a dry gas flow rate of 8 L/min on a QTOF Bruker Impact II system (Bruker Daltonics, Germany). The Compass data analysis tool (V. 4.4.110) was used to analyze the liquid chromatography–MS data. The column flow rate was set at 0.3 mL/min, the draw speed was set at 2 µL/s, and the column oven temp was 35 °C, with a total injection volume of 2 µL. The mass spectrometer (MS) settings were as follows: drying gas flow of 8 L/min, collision energy of 7.0 eV, capillary voltage of 4500 V, ionization energy of 4.0 eV, gas temperature of 220 °C, and cycle time of 0.5 s. Data analysis was performed using Bruker Compass Data Analysis 4.3 (Bruker Daltonik, Germany, 2014), and results were compared with the NIST (National Institute of Standards and Technology, 2005) library. Peak identification was based on mass, MS/MS, and retention time (r.t.), with accurate mass and MS/MS spectral data being cross-referenced against the PubChem, Kyoto Encyclopedia of Genes and Genomes (KEGG), MetFrag, ChemSpider, and Chemical Entities of Biological Interest (ChEBI) databases. The definitive identities of the secondary metabolites were confirmed with a precursor ion at a particular “*m*/*z*” value and the MS/MS analysis at a definitive “*m*/*z*” value. For instance, the compound “Fecosterol” was confirmed with a precursor ion at “*m*/*z”* 399.365, and the MS/MS analysis revealed key fragment ions at “*m*/*z*” 381 (loss of water) and “*m*/*z”* 149, which is consistent with known fragmentation patterns for sterols. The fragmentation patterns for each compound were compared with databases. Similarly, the identities of other metabolites were confirmed in this study.

### 3.10. Data Analysis

The data obtained in this study were analyzed and visualized in R studio 4.3.3 [94] and with Microsoft Excel 2021 Package. A specialized R package (*pheatmap*) was employed for the visualization of crude secondary metabolite data and the positive control [95]. All experiments were performed in replicates as indicated, and results are expressed as mean ± standard deviation.

## 4. Conclusions

In this study, we demonstrated the significant antibacterial potential of secondary metabolites from selected endophytes of *S. mauritianum*. The identification of bioactive compounds across the three endophytes, *P. chrysogenum*, *Fusarium* sp., and *P. leucadendri*, highlights the diversity and potency of these natural products. Notably, all three endophytes exhibited significant antibacterial activity with the MICs of their crude extracts ranging from 0.02 to 4 mg/mL (for active extracts), emphasizing their promise for further exploration in the development of novel antimicrobial agents. The findings accentuate the role of endophytes as a sustainable and valuable resource in addressing the global challenge of AMR.

## Figures and Tables

**Table 1:** Isolation, distribution, and total secondary metabolites of fungal endophytes in *S. mauritianum* plant parts.

Accession No.	Fungal Endophyte	Stem	Leaves	Fruits	Total Secondary Metabolites
MF928767	*Paracamarosporium leucadendri*	−	−	+	29
MF928763	*Fusarium* sp.	−	+	+	23
MF928764	*Penicillium chrysogenum*	−	+	+	11

Legend: + = present; − = absent.

**Table 2:** Identified secondary metabolites from *Paracamarosporium leucadendri* isolated from *S. mauritianum*.

S/N	Compound Name	Retention Time	*m*/*z*	Peak Area	Chemical Formula
1	*N*-Methyl-l-proline (peptide)	1.45	130.086	32,280	C_6_H_11_N_1_O_2_
2	Indan-1-ol	1.21	135.08	24,201	C_9_H_10_O_1_
3	Fecosterol	4.89	399.365	11,312	C_28_H_46_O_1_
4	Phyllanthin	4.9	419.246	28,061	C_24_H_34_O_6_
5	Glutathione amide disulfide	4.9	611.187	28,061	C_20_H_34_N_8_O_10_S_2_
6	Tris (2,4-ditert-butyl phenyl) phosphate	10.39	663.455	28,061	C_42_H_63_O_4_P_1_
7	Irganox 858	10.39	664.46	11,633	C_39_H_61_N_5_O_2_S_1_
8	Aniracetam	10.65	220.098	13,235	C_12_H_13_N_1_O_3_
9	9-Octadecenamide, n-butyl-	10.69	338.343	13,012	C_22_H_43_N_1_O_1_
10	Bicyclo [2.2.1] hept-2-en-7-ol	11.17	111.081	33,799	C_7_H_10_O_1_
11	Bellendine	11.07	206.119	33,799	C_12_H_15_N_1_O_2_
12	4-Vinylguaiacol	11.45	151.076	116,589	C_9_H_10_O_2_
13	Caffeine	11.45	195.089	116,589	C_8_H_10_N_4_O_2_
14	Solavetivone	11.45	219.175	116,589	C_15_H_22_O_1_
15	Dehydrovomifoliol	11.45	223.132	116,589	C_13_H_18_O_3_
16	5-Hydroxy-alpha-gurjunene	11.45	223.205	116,589	C_15_H_26_O_1_
17	Pirimicarb	11.45	239.15	116,589	C_11_H_18_N_4_O_2_
18	α-Eleostearic acid	11.45	279.233	116,589	C_18_H_30_O_2_
19	Thiamine acetic acid (vitamin B)	11.45	280.098	116,589	C_12_H_15_N_4_O_2_S_1_
20	Xanomeline	11.45	282.161	116,589	C_14_H_23_N_3_O_1_S_1_
21	Tridihexethyl	11.45	319.286	116,589	C_21_H_36_N_1_O_1_
22	Ethyl butylacetylaminopropionate	11.82	216.16	54,174	C_11_H_21_N_1_O_3_
23	Felbamate	11.82	239.101	54,174	C_11_H_14_N_2_O_4_
24	L-Cladinose	3.15	177.1117	0	C_8_H_16_O_4_
25	Albaflavenone	10.65	219.1753	13,235	C_15_H_22_O_1_
26	Carvacrol	11.45	151.112	116,589	C_10_H_14_O_1_
27	6-*cis*-docosenamide	11.45	338.343	116,589	C_22_H_43_N_1_O_1_
28	Seiricardine A	11.82	239.1983	54,174	C_15_H_26_O_2_
29	Rishitin	11.45	223.1693	116,589	C_14_H_22_O_2_

**Table 3:** Identified secondary metabolites from *Fusarium* sp. isolated from *S. mauritianum*.

S/N	Compound Name	Retention Time	*m*/*z*	Peak Area	Chemical Formula
1	3-Aminophenol	0.67	110.7653	20,855.62	C_6_H_7_N_1_O_1_
2	Aminohydroquinone	0.68	126.0548	163,862.47	C_6_H_7_N_1_O_2_
3	Benzocaine	0.76	166.0849	1325.77	C_9_H_11_N_1_O_2_
4	Methyldopa	0.76	212.0923	1325.77	C_10_H_13_N_1_O_4_
5	Nicotinamide (vitamin B3)	0.81	124.0393	102,375.62	C_6_H_5_N_1_O_2_
6	Actinidine	0.84	148.1118	102,375.62	C_10_H_13_N_1_
7	1-phenyl pyridine-1-ium	0.93	157.0892	112,684.58	C_11_H_10_N_1_
8	2-[tert-butyl(methyl) amino] ethanethiol	0.98	148.1142	61,849.06	C_7_H_17_N_1_S_1_
9	Zeatin (cytokinins)	0.98	220.1184	5196.3	C_10_H_13_N_5_O_1_
10	ethyl 4-amino-1*H*-pyrrole-2-carboxylate	1.01	155.0817	231,881.42	C_7_H_10_N_2_O_2_
11	Norselegiline	3.26	174.1283	10,494.4	C_12_H_15_N_1_
12	Pifithrin β	4.81	269.1121	3,211,781.75	C_16_H_16_N_2_S_1_
13	Capsi-amide	4.81	270.2803	32,11,781.75	C_17_H_35_N_1_O_1_
14	Montanol	4.81	353.2666	32,664.74	C_21_H_36_O_4_
15	16-methyl-1-morpholin-4-ylheptadecan-1-one	4.81	354.3363	32,664.74	88.0757
16	1,4,8,11-tetraethyl-1,4,8,11-tetrazacyclotetradecane	10.03	313.3309	2224.51	C_18_H_40_N_4_
17	Tridemorph	11.79	298.3117	41,864.85	C_19_H_39_N_1_O_1_
18	Messagenin	12.16	445.3695	6640.07	C_29_H_48_O_3_
19	3-allylthiophene	0.81	125.0432	102,375.62	C_7_H_8_S_1_
20	2-nitroimidazo[2,1-b][1,3]oxazole	1.01	154.0243	231,881.42	C_5_H_3_N_3_O_3_
21	Withasomnine	4.83	185.1073	42,543.51	C_12_H_12_N_2_
22	1-(4-methyl-1-piperidinyl)-2-tetra decanol	10.03	312.3281	2224.51	C_20_H_41_N_1_O_1_
23	4-dodecyl morpholine	10.03	256.2647	25,182.35	C_16_H_33_N_1_O_1_

**Table 4:** Identified secondary metabolites from *Penicillium chrysogenum* isolated from *S. mauritianum*.

S/N	Compound Name	Retention Time	*m*/*z*	Peak Area	Chemical Formula
1	Metoprolol	6.41	268.1927	1627.21	C_15_H_25_N_1_O_3_
2	Petromyzonol	11.24	395.3127	10,748.93	C_24_H_42_O_4_
3	6-Ethylchenodeoxycholic acid	11.27	421.3287	5593.51	C_26_H_44_O_4_
4	Methamphetamine	11.4	150.1276	17,864.26	C_10_H_15_N_1_
5	Phenethylamine	12.05	122.096	800.37	C_8_H_11_N_1_
6	Amphetamine	13.01	136.1117	1399.35	C_9_H_13_N_1_
7	Tubulysin B	9.87	830.4306	1840.95	C_42_H_63_N_5_O_10_S_1_
8	Azafrin	8.71	427.2811	14,967.18	C_27_H_38_O_4_
9	Dihydro-beta-erythroidine (nicotine)	9.45	276.1546	13,381.62	C_16_H_21_N_1_O_3_
10	Ochrolifuanine A	8.59	439.2818	19,935	C_29_H_34_N_4_
11	1,8-Diazacyclotetradecane-2,9-dione	3.51	227.1753	571.73	C_12_H_22_N_2_O_2_

## Data Availability

Data are contained within the article and Supplementary Materials.

## Supplementary Materials

The following supporting information can be downloaded at https://www.mdpi.com/article/10.3390/molecules29204924/s1, Supplementary File S1: Raw spectral data of metabolites from *Paracamarosporium leucadendri.* Supplementary File S2: Raw spectral data of secondary metabolites Identified from *Fusarium* sp. solated from *S. mauritianum.* Supplementary File S3: Raw spectral data of secondary metabolites Identified from *Penicillium chrysogenum* isolated from *S. mauritianum.*
