# Supplementary material for: Identification and Assessment of Secondary Metabolites from Three Fungal Endophytes of Solanum mauritianum Against Public Health Pathogens
Source: Molecules. 2024 Oct 17;29(20):4924. doi: 10.3390/molecules29204924 (PMC11510704; doi:10.3390/molecules29204924)
Supplement: Supplementary file 1 [file molecules-29-04924-s001.zip › molecules-3241240-supplementary.pdf]

SUPPLEMENTARY MATERIALS

Supplementary file S1: Raw spectral data of metabolites from *Paracamarosporium leucadendri* (Table S1 of the manuscript).

S/N 1 & 2.

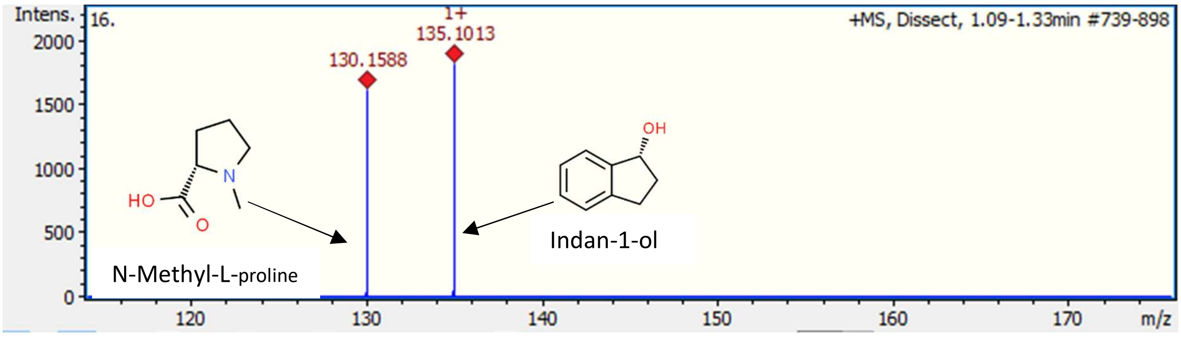

S/N 3

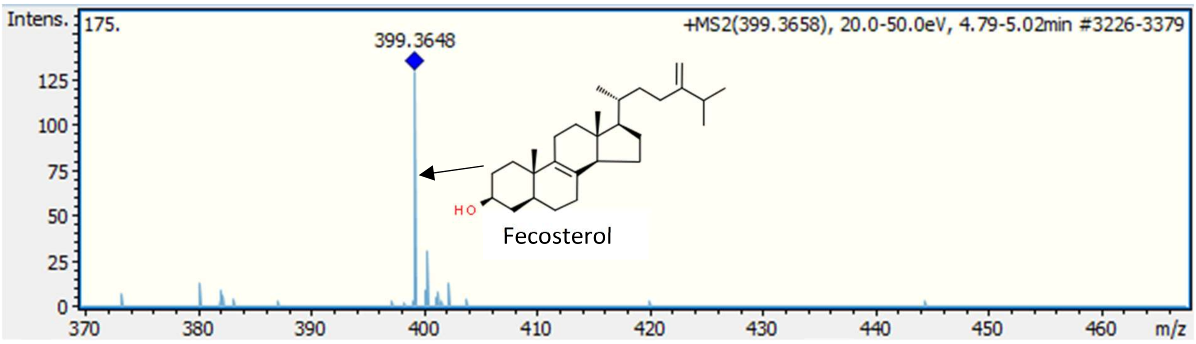

S/N 4

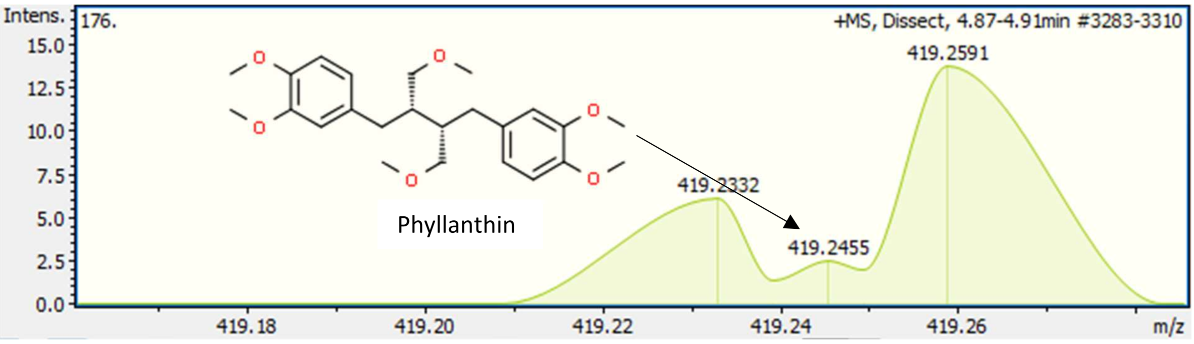

S/N 5

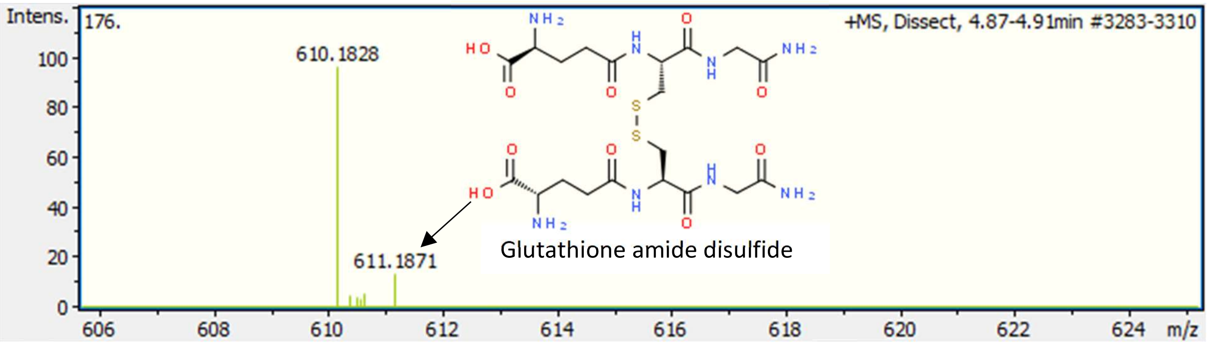

S/N 6

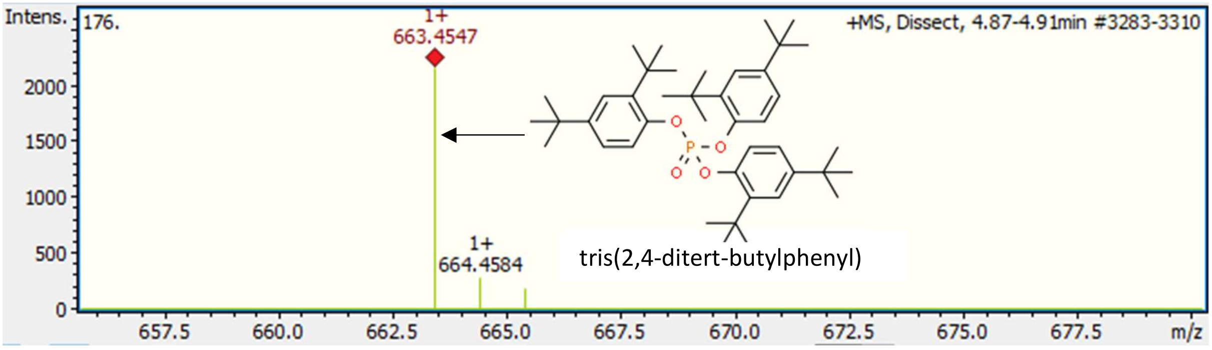

S/N 7

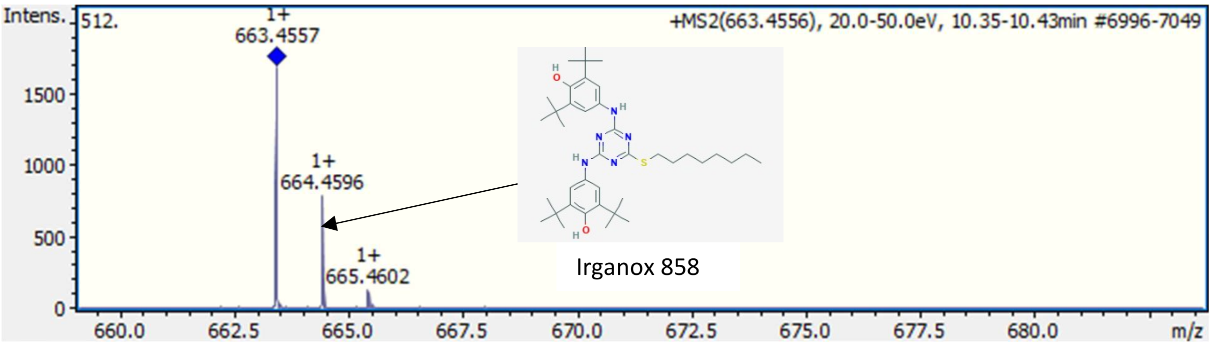

S/N 8

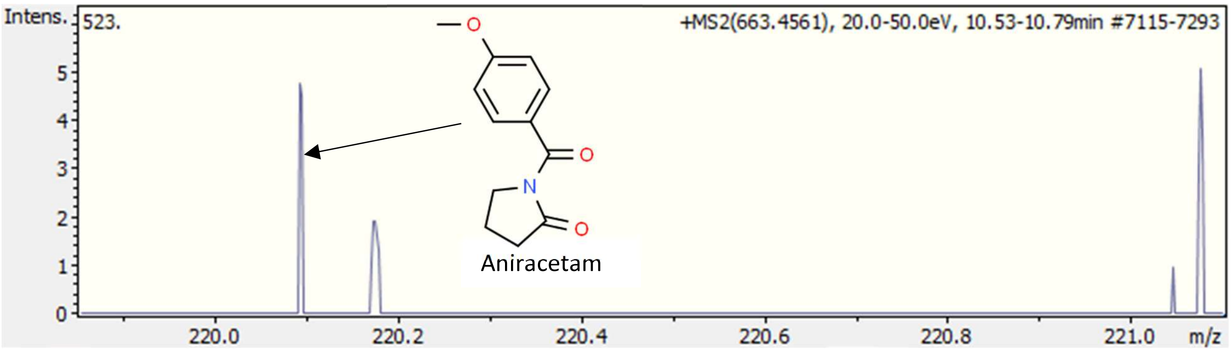

S/N 9

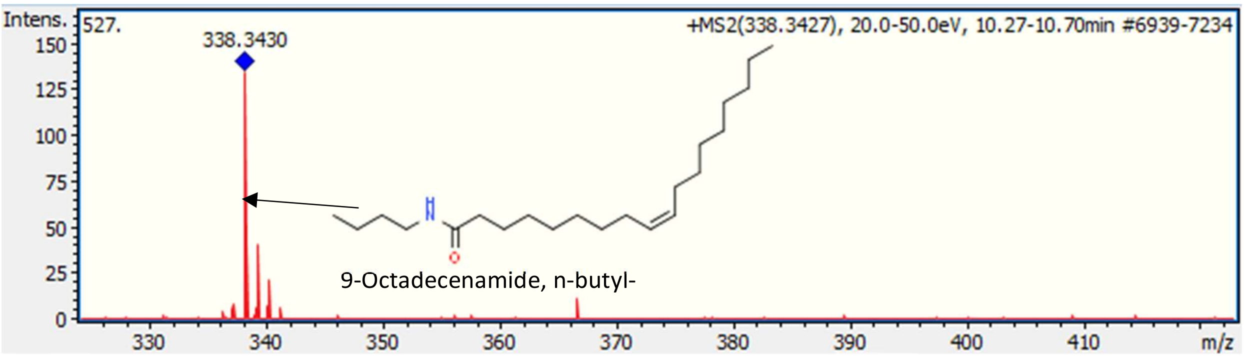

S/N 10

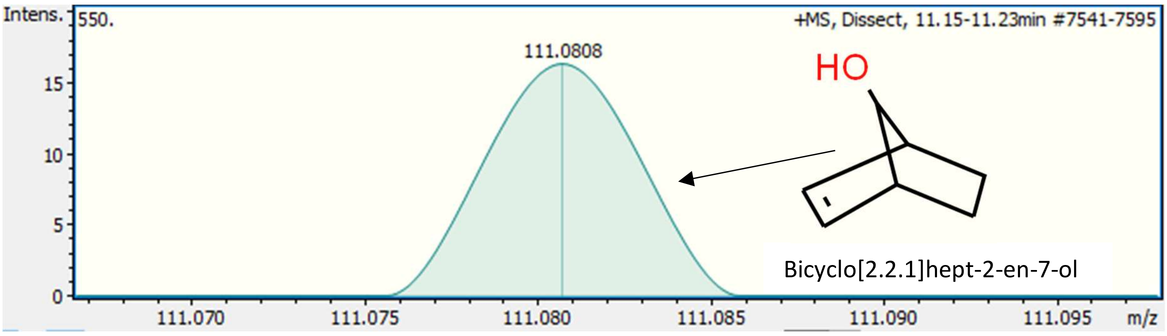

S/N 11

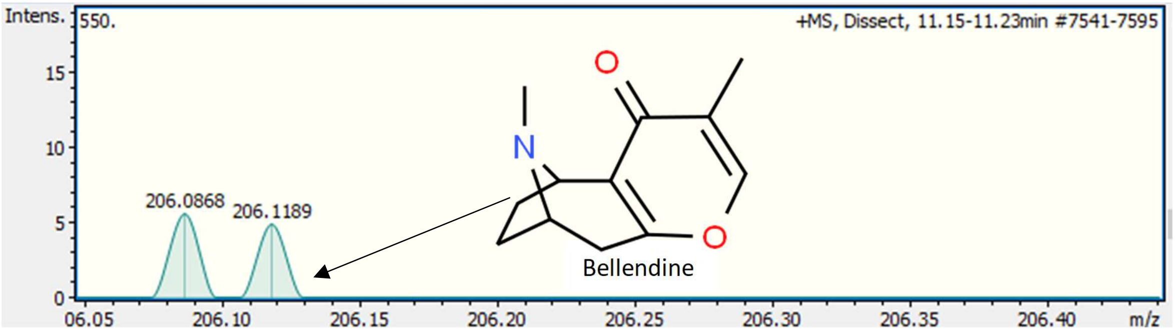

S/N 12

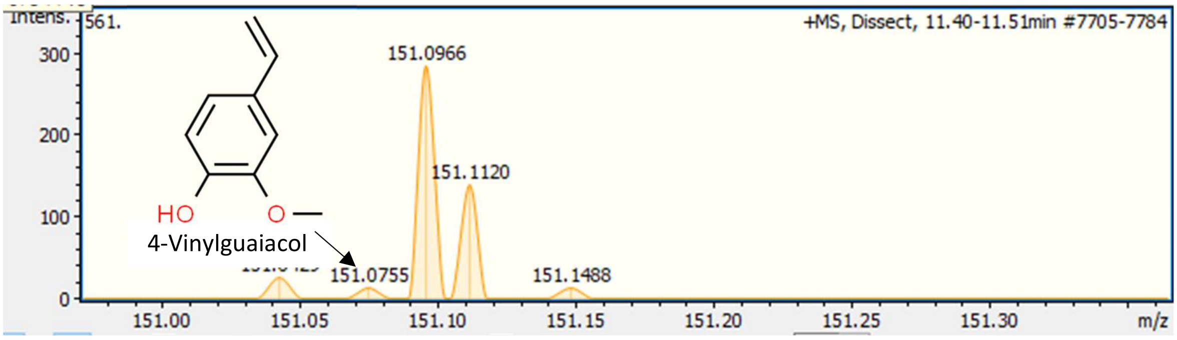

S/N 13

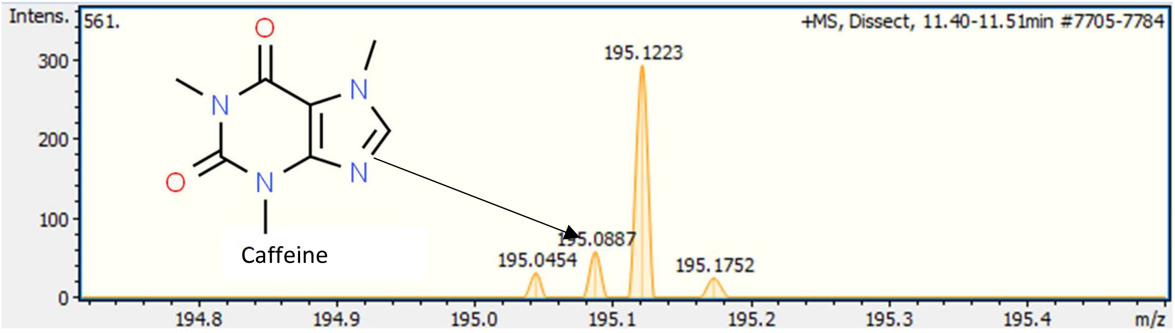

S/N 14

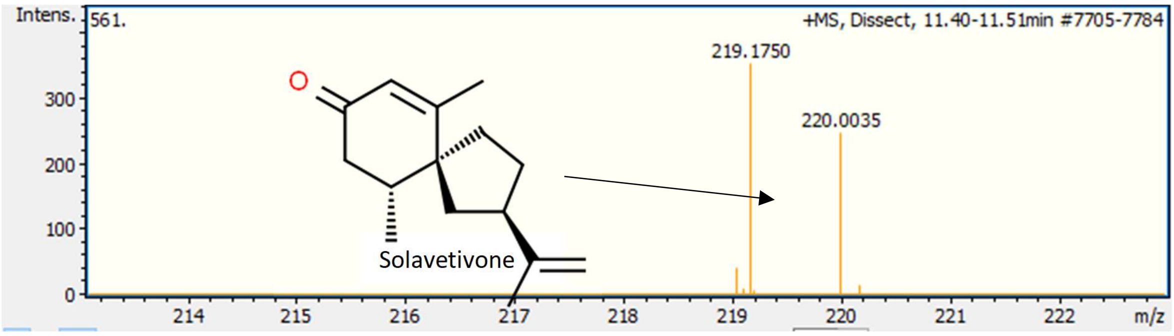

S/N 15

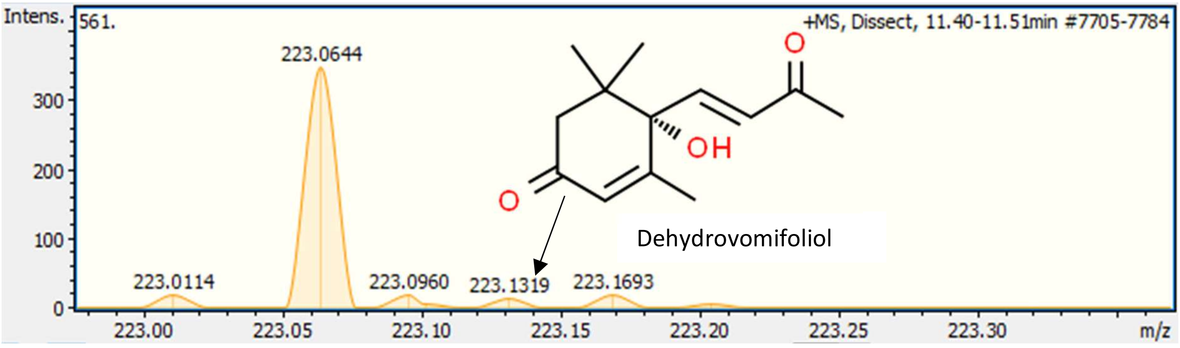

S/N 16

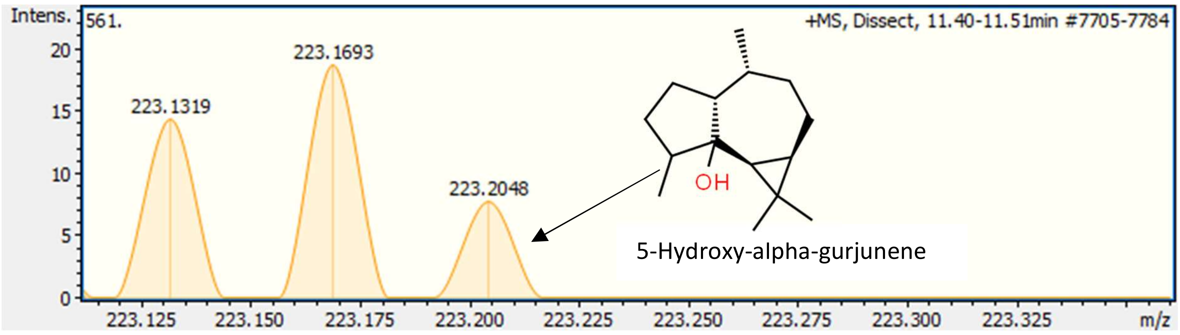

S/N 17

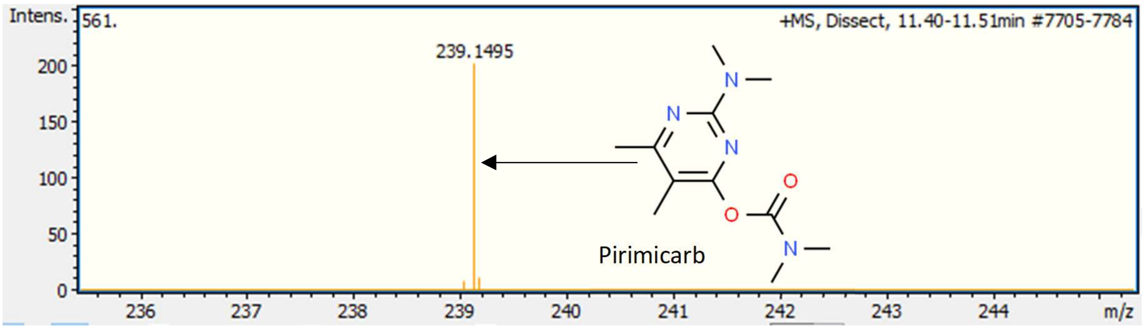

S/N 18

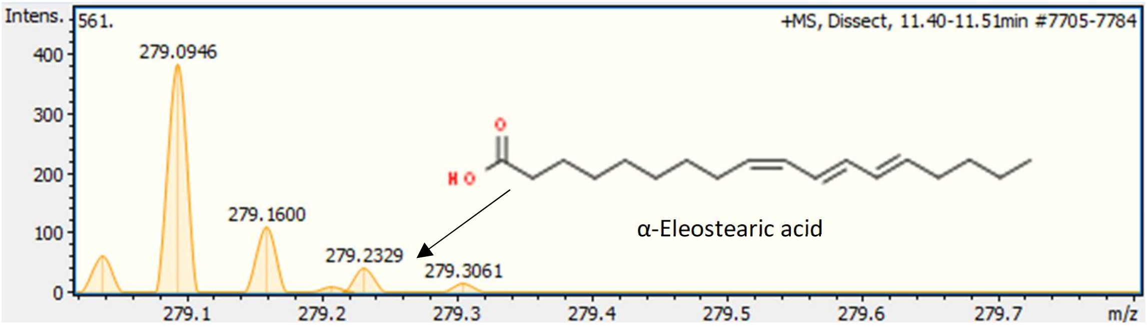

S/N 19

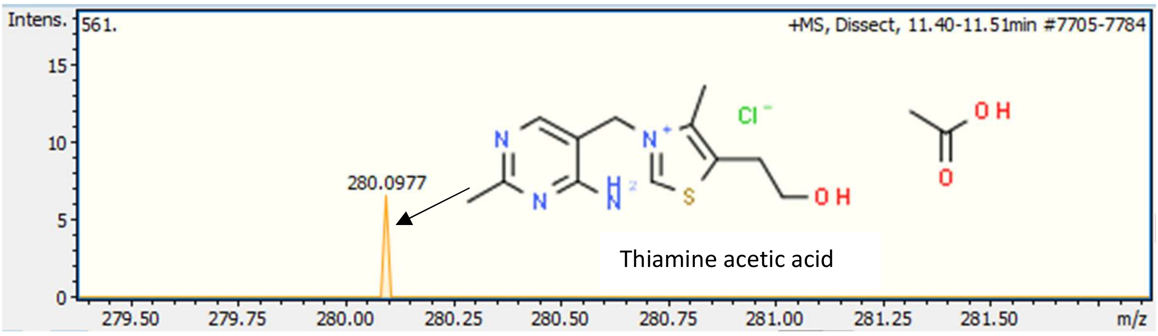

S/N 20

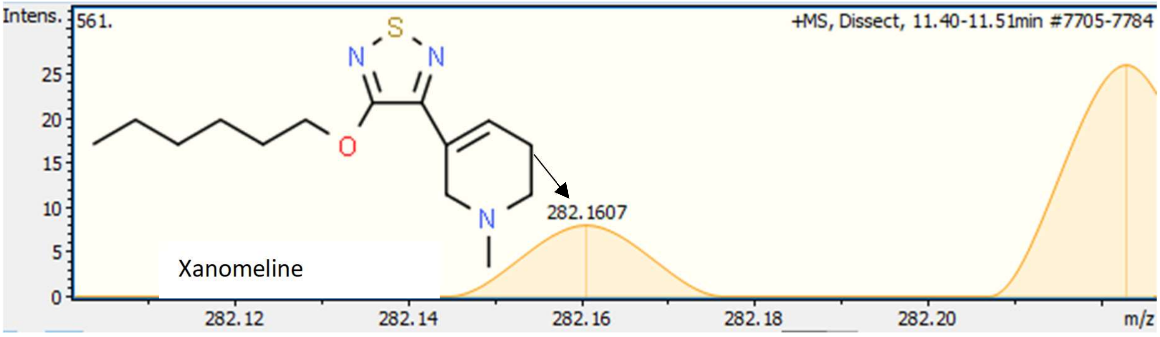

S/N 21

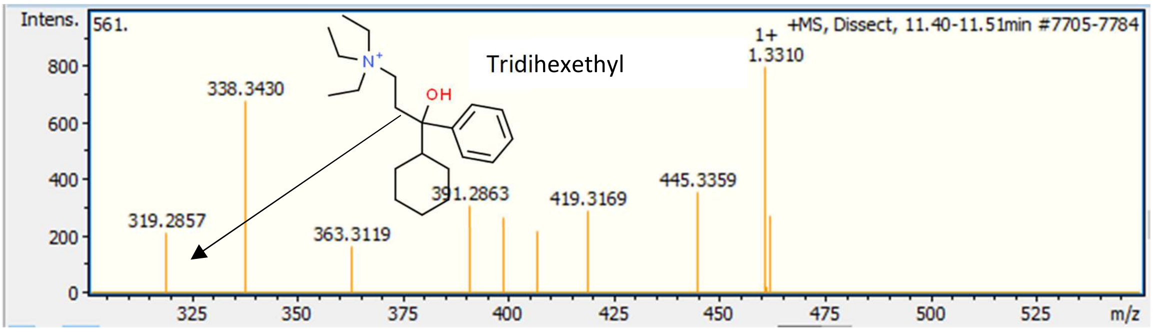

S/N 22

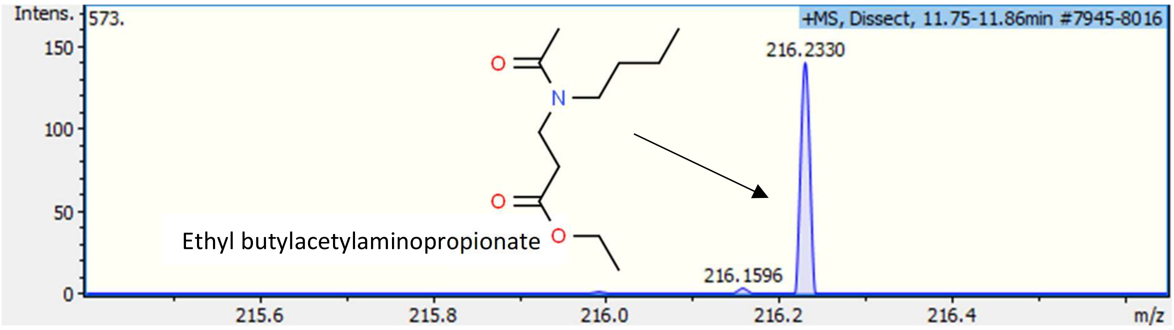

S/N 23

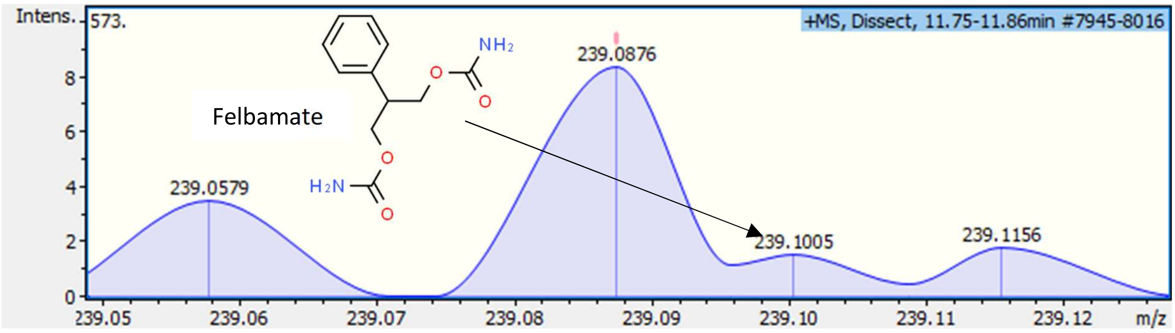

S/N 24

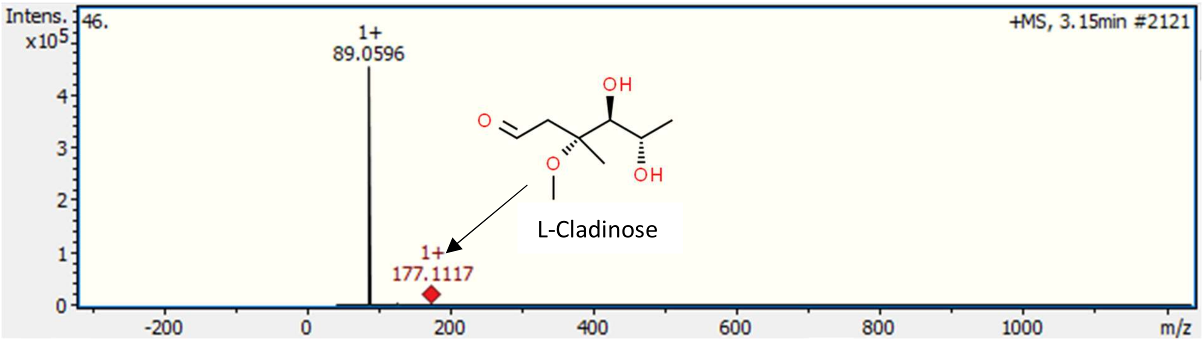

S/N 25

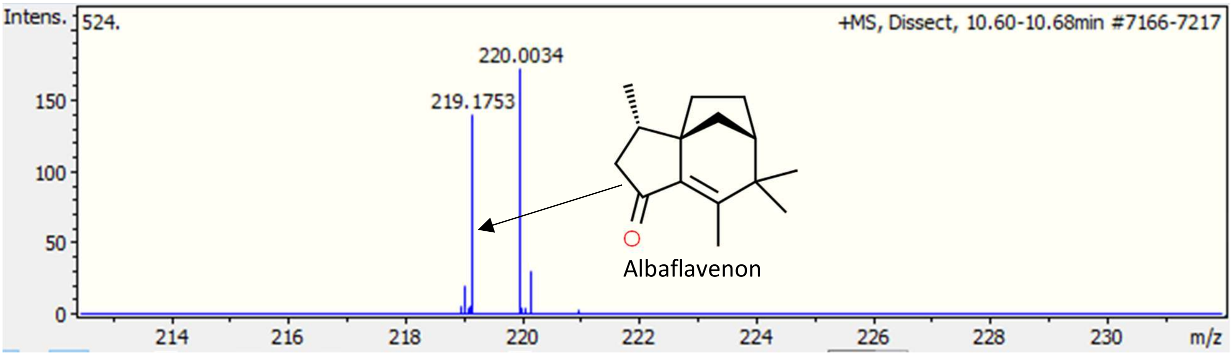

S/N 26

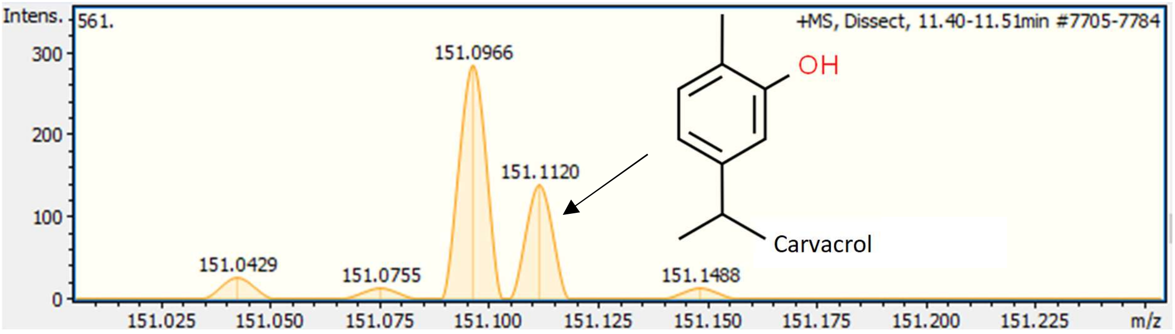

S/N 27

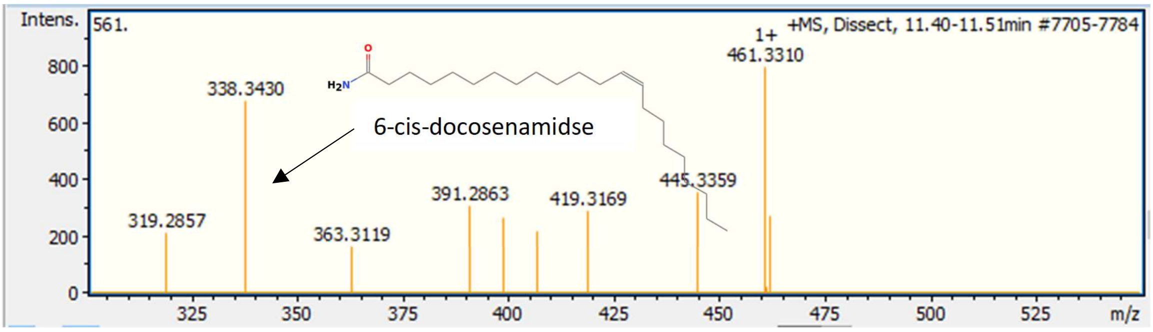

S/N 28

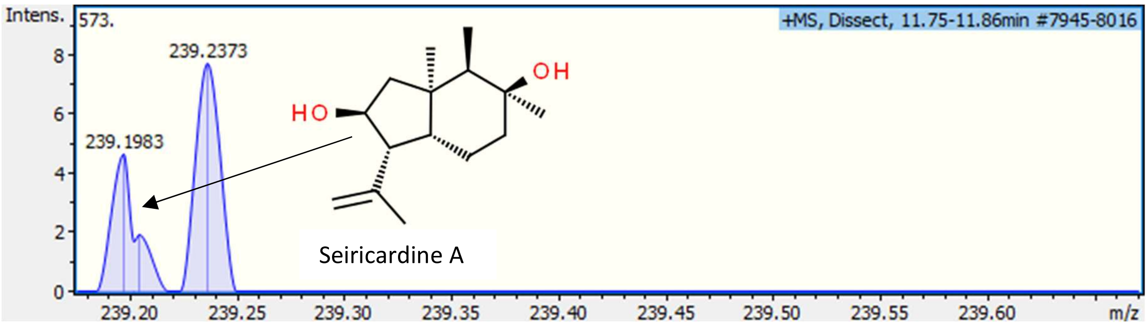

S/N 29

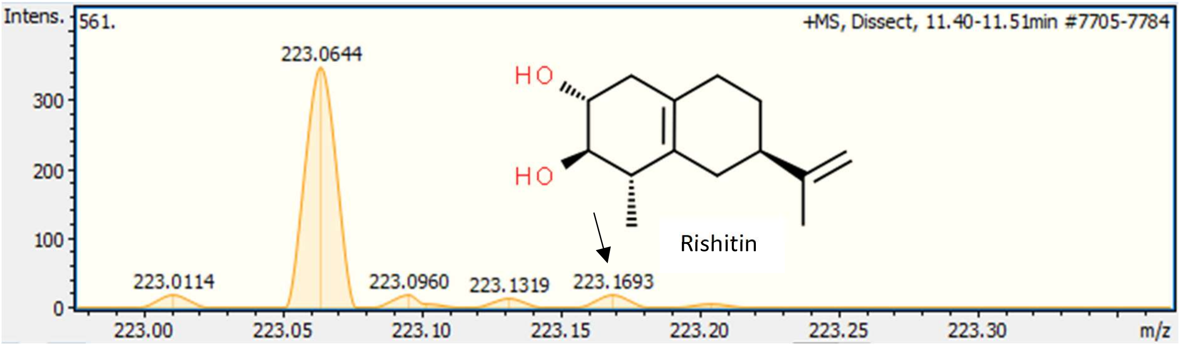

Supplementary file 2 of Raw spectral data of secondary metabolites Identified from *Fusarium* sp. isolated from *S. mauritianum* (From Table 2)

S/N 1b

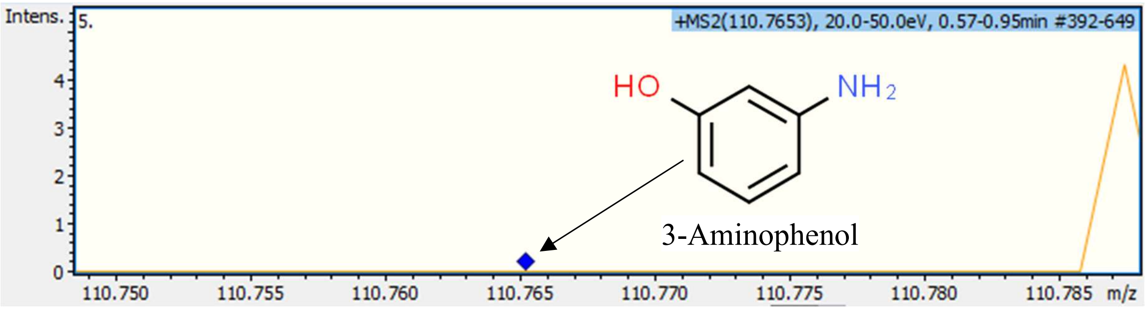

S/N 2b

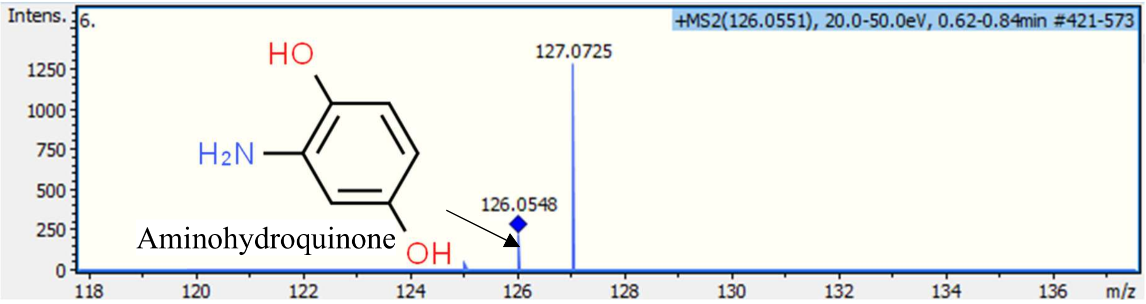

S/N 3b

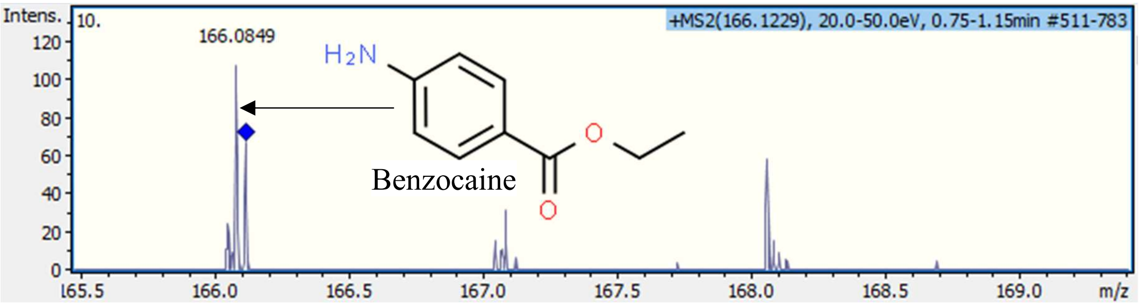

S/N 4b

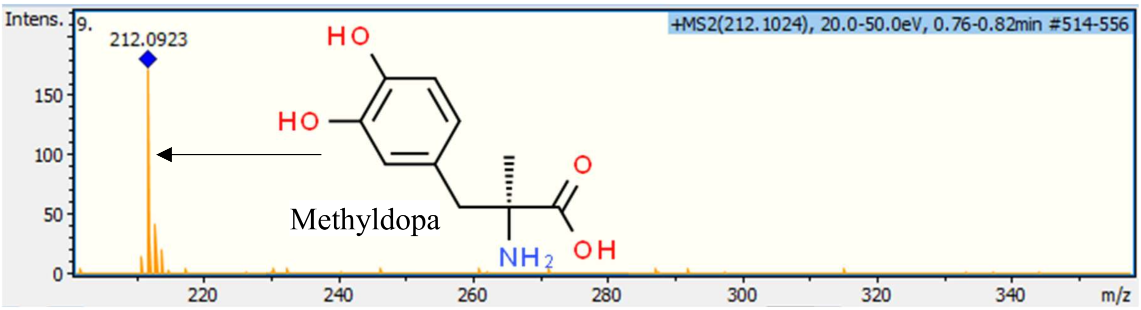

S/N 5b

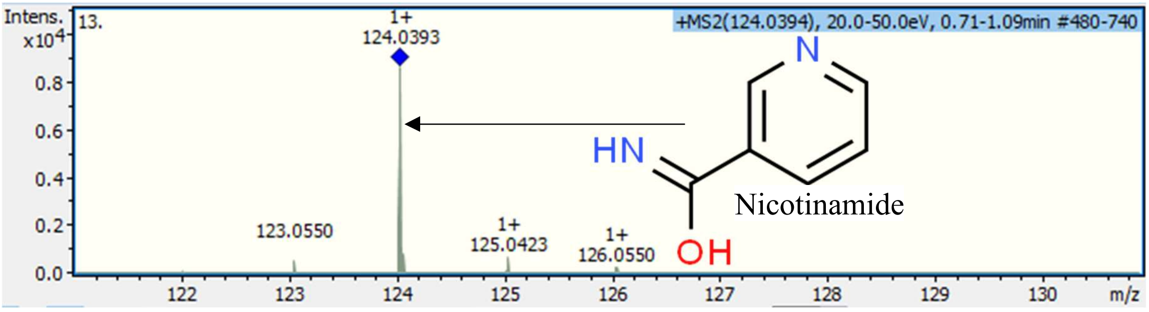

S/N 6b

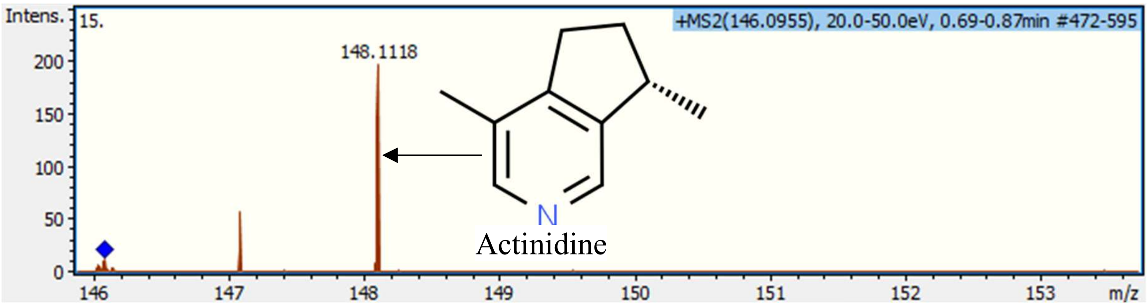

S/N 7b

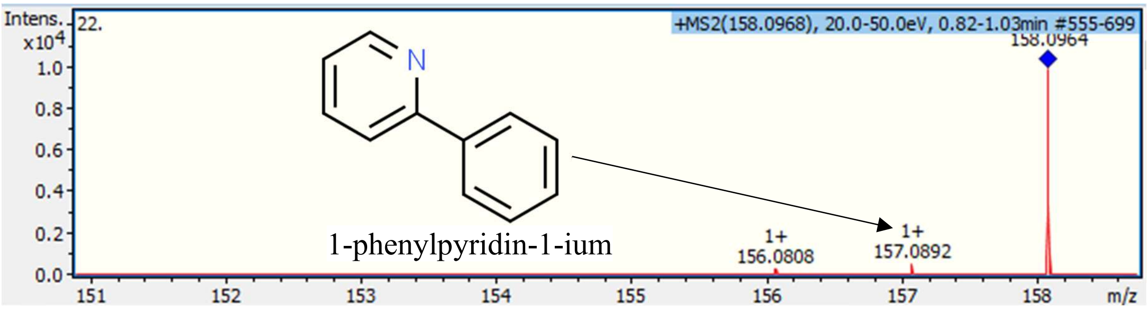

S/N 8b

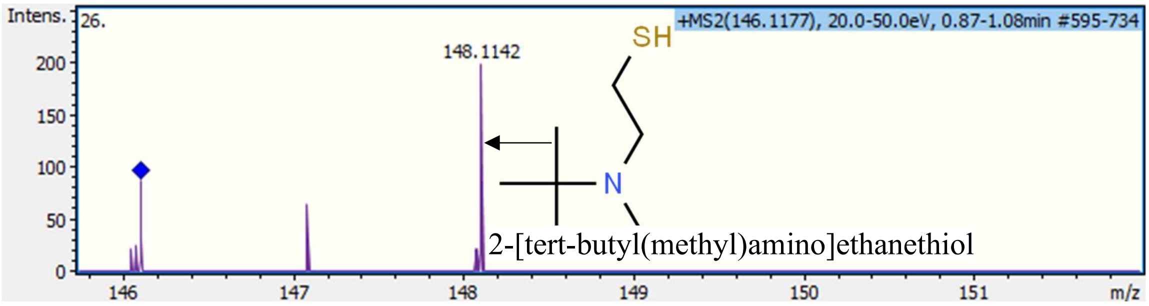

S/N 9b

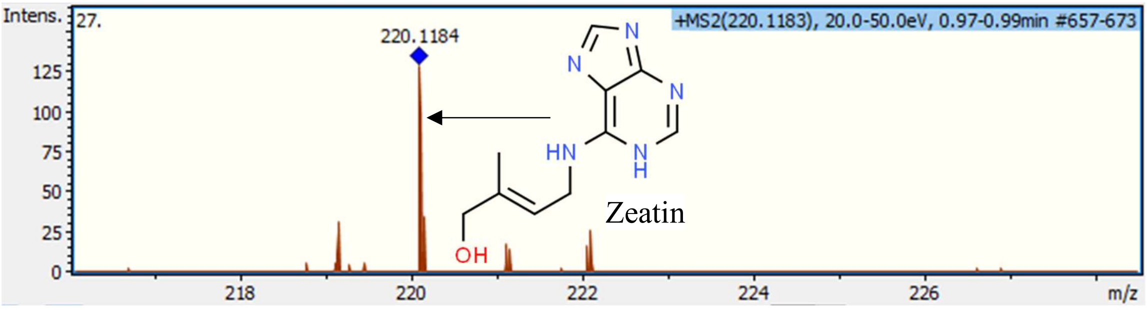

S/N 10b

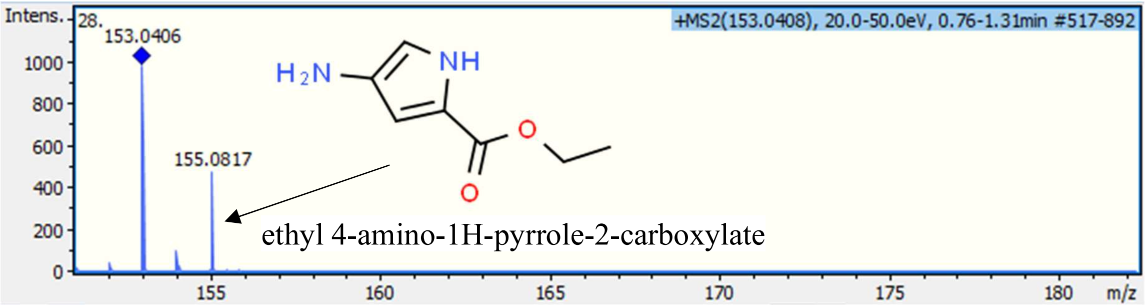

S/N 11b

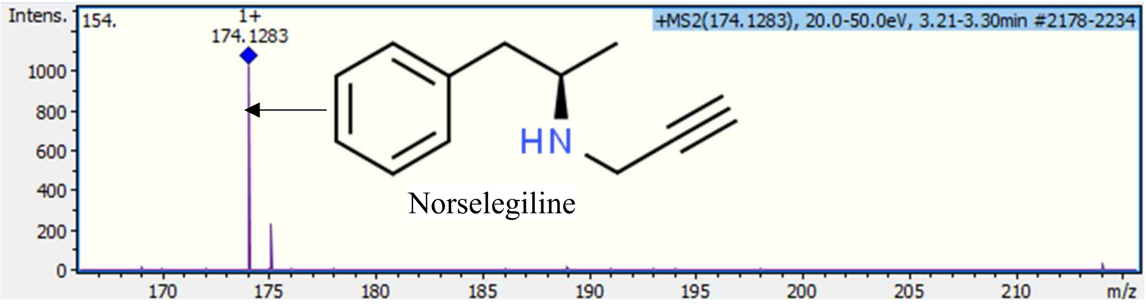

S/N 12b

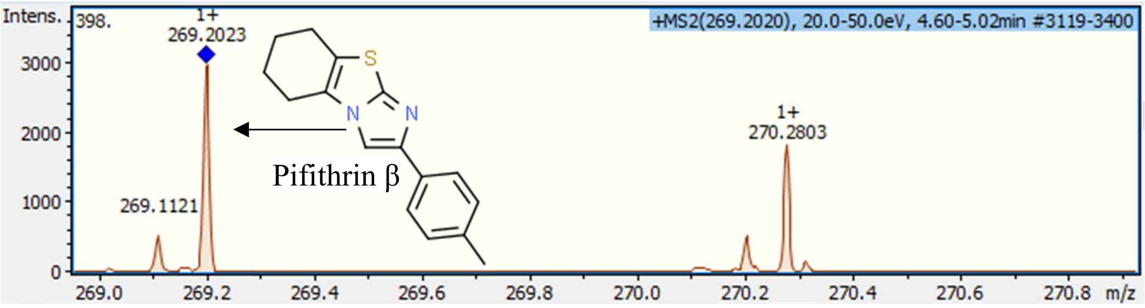

S/N 13b

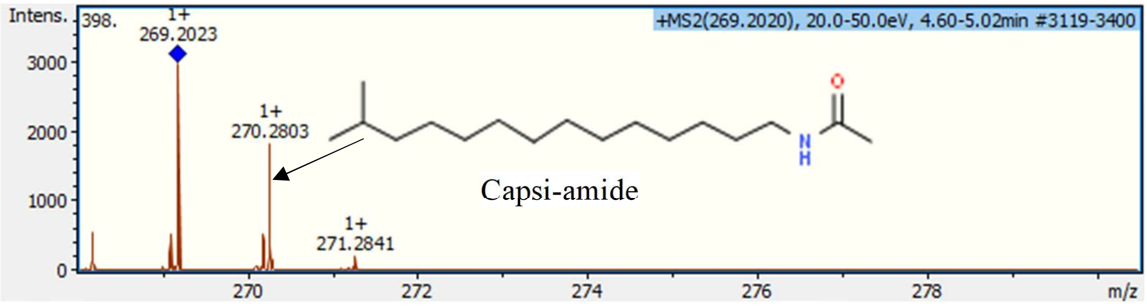

S/N 14b

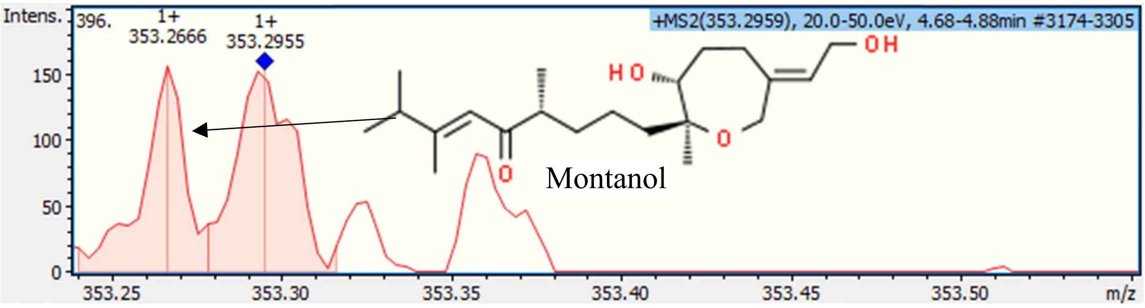

S/N 15b

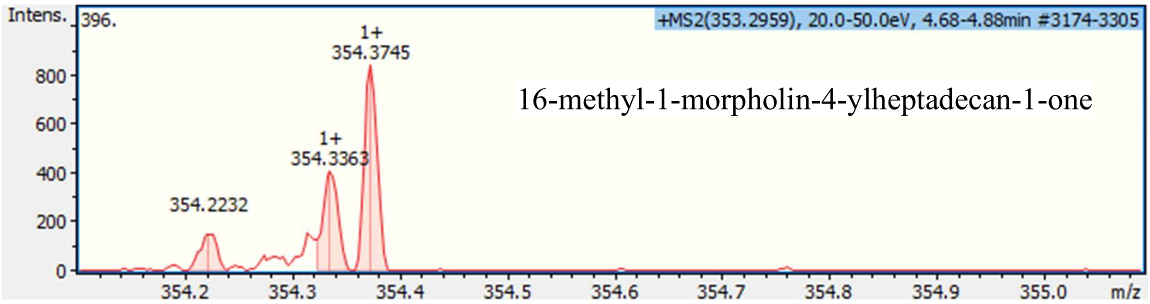

S/N 16b

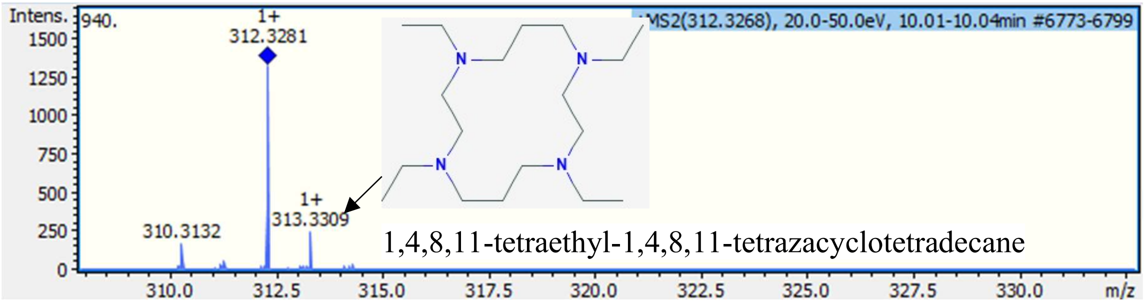

S/N 17b

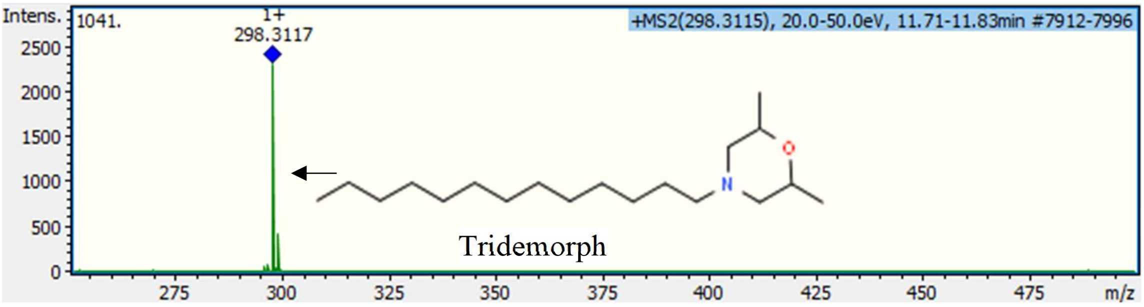

S/N 18b

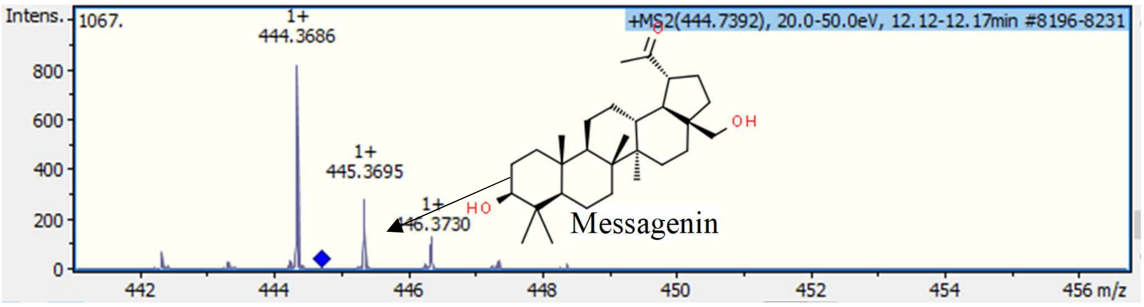

S/N 19b

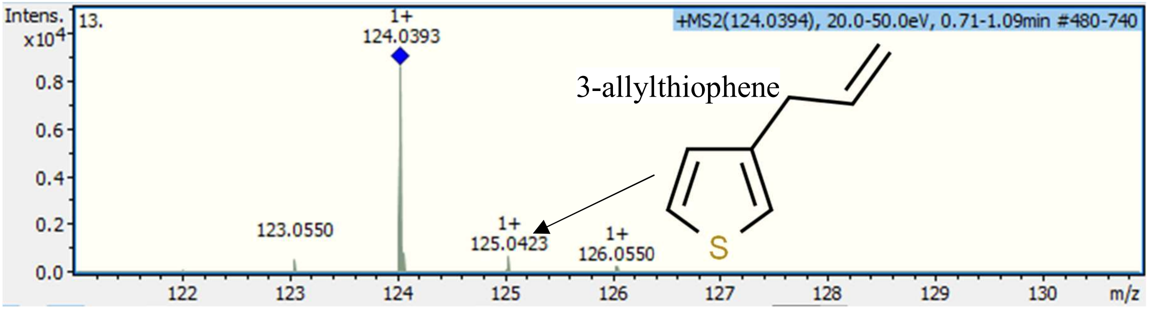

S/N 20b

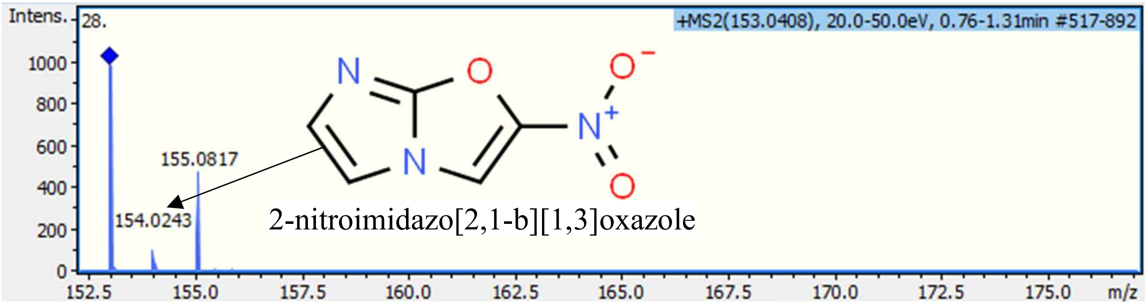

S/N 21b

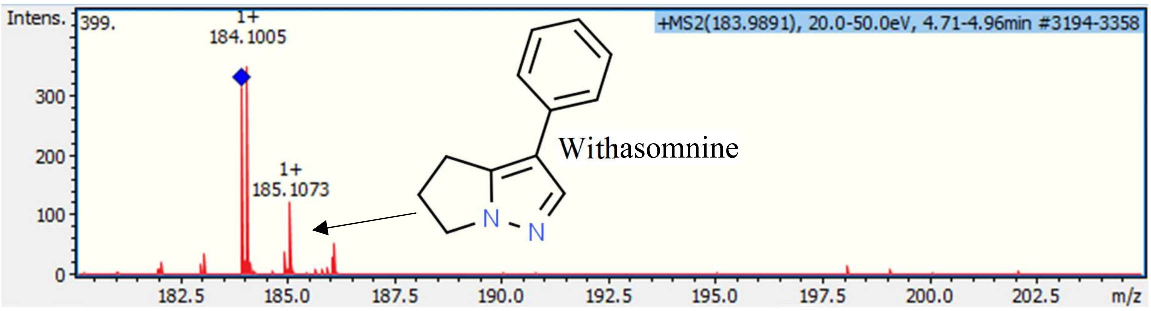

S/N 22b

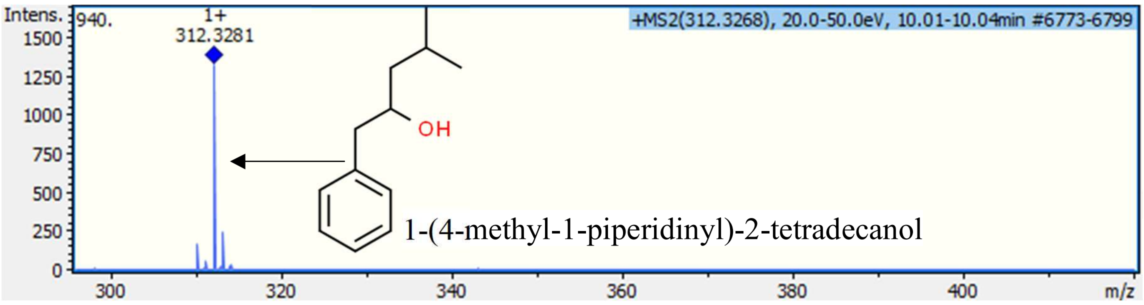

S/N 23b

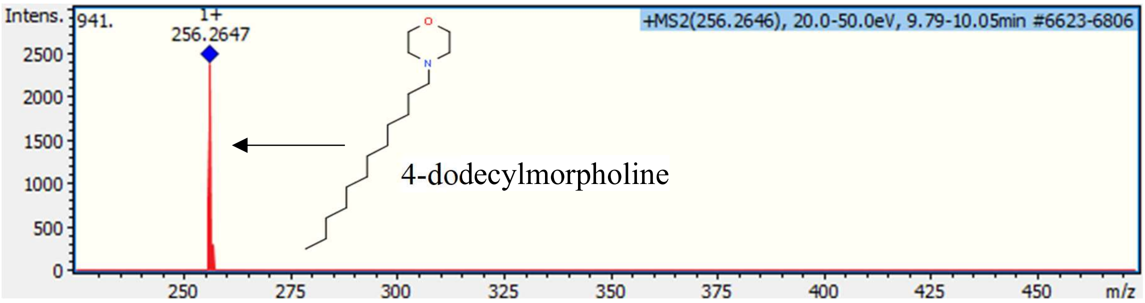

Supplementary file 3 of Raw spectral data of secondary metabolites Identified from *Penicillium chrysogenum* isolated from *S. mauritianum* (From Table 3)

S/N 1c

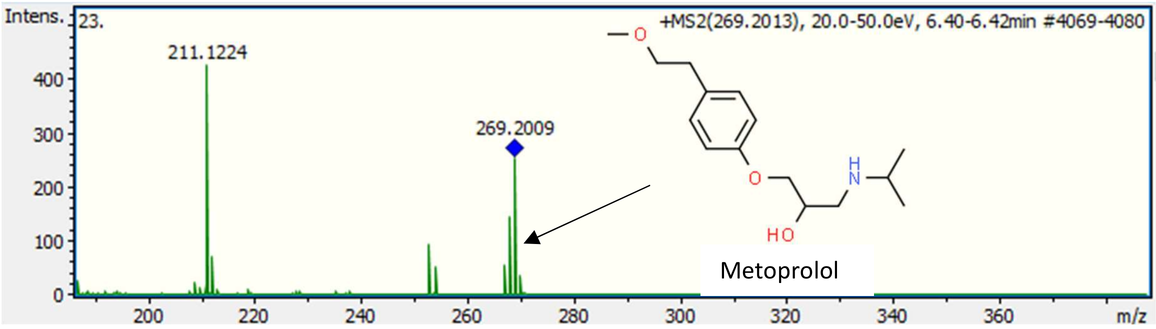

S/N 2c

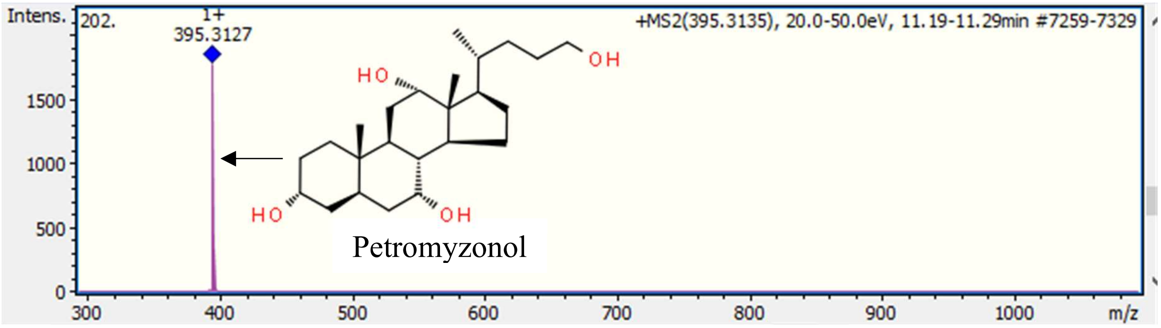

S/N 3c

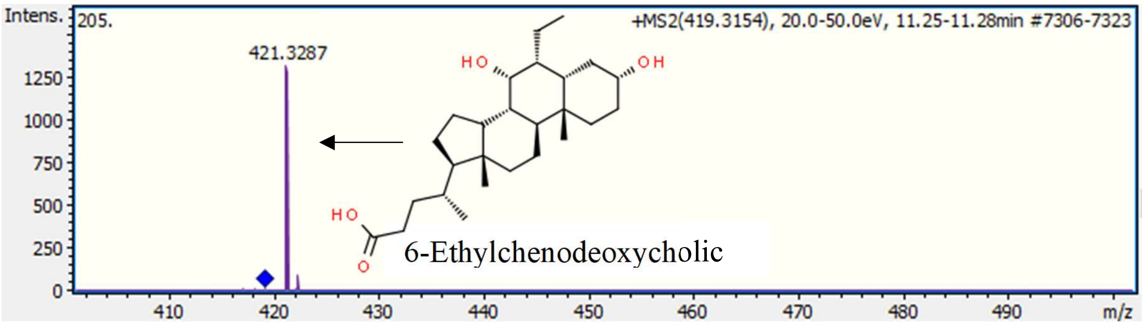

S/N 4c

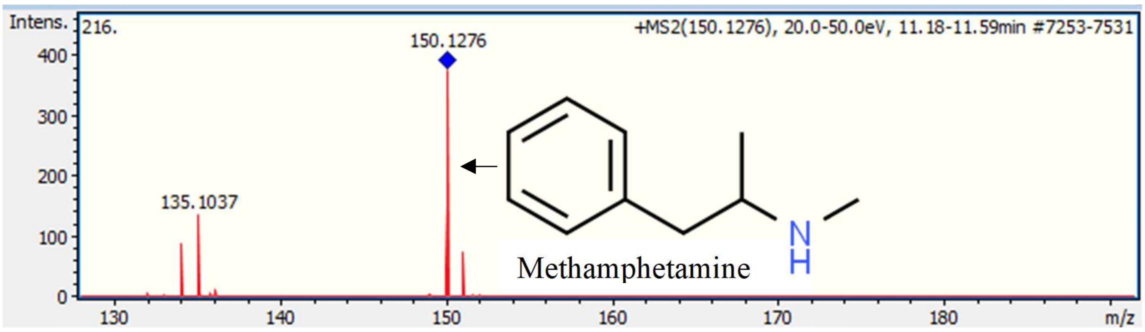

S/N 5c

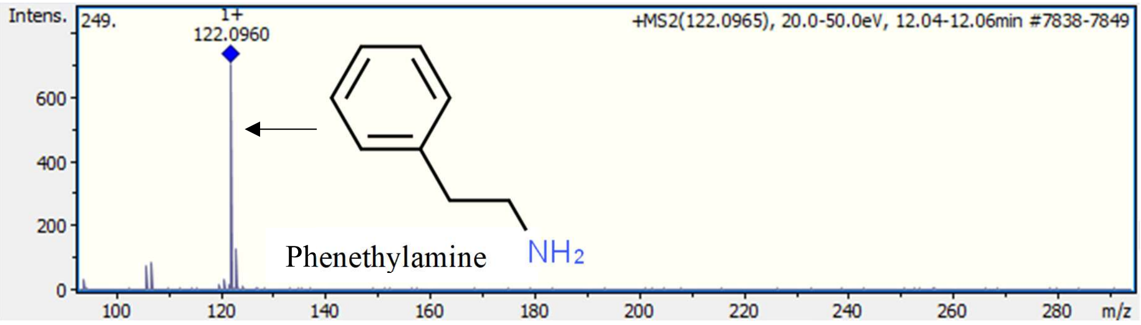

S/N 6c & 7c

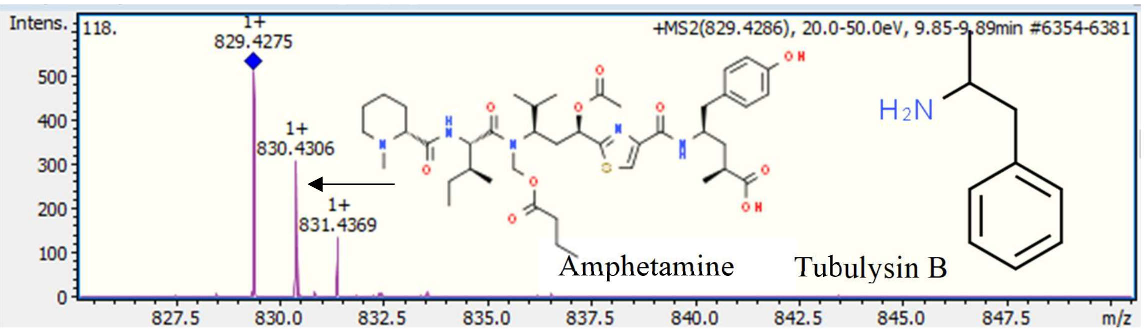

S/N 8c

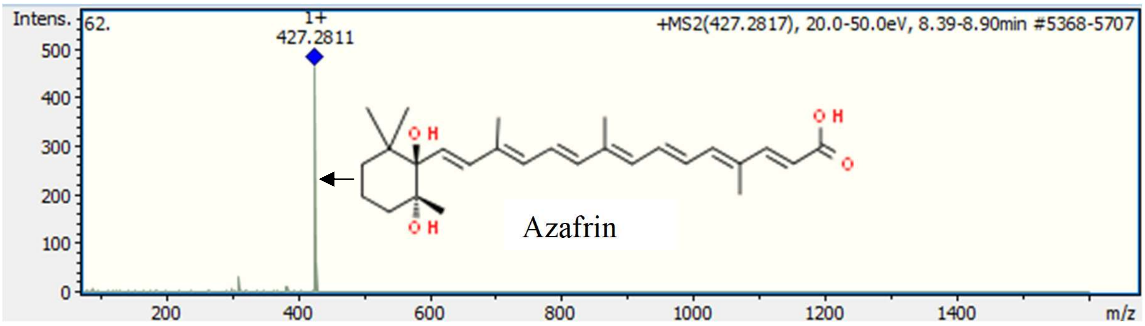

S/N 9c

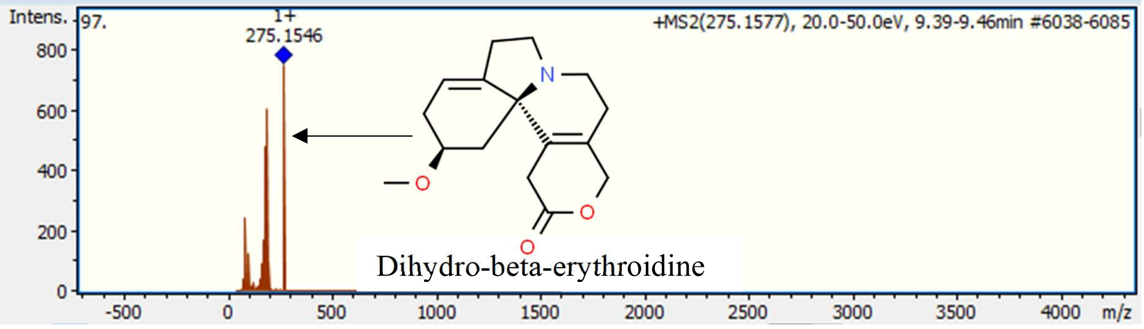

S/N 10c

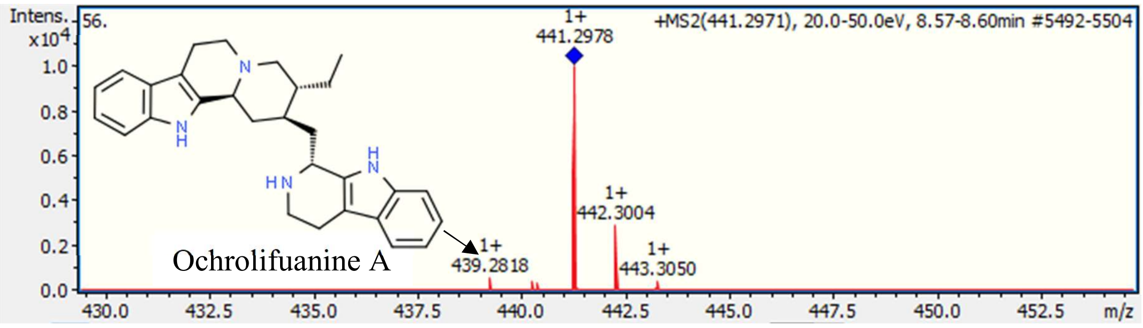
Intens.
x10^4
56.
+MS2(441.2971), 20.0-50.0eV, 8.57-8.60min #5492-5504
1+
441.2978
1+
442.3004
1+
443.3050
1+
439.2818
Ochrolifuanine A
m/z

S/N 11c

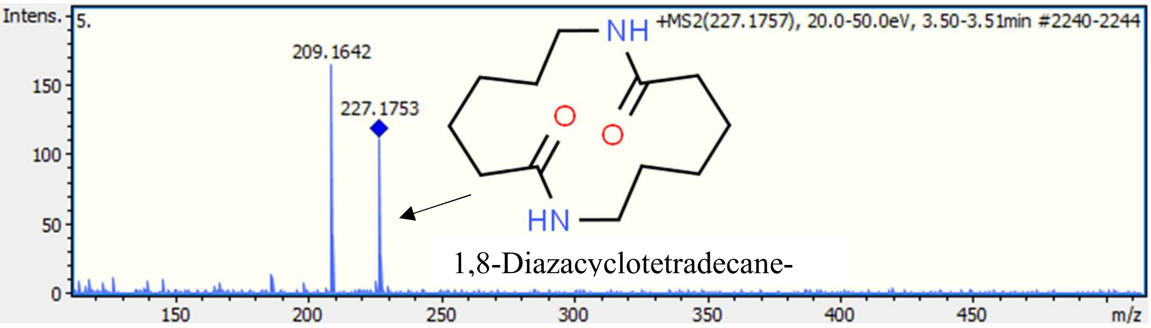
Intens.
5.
+MS2(227.1757), 20.0-50.0eV, 3.50-3.51min #2240-2244
209.1642
227.1753
1,8-Diazacyclotetradecane-
m/z
